# In Vitro Investigation of the Interaction of Tolbutamide and Losartan with Human Serum Albumin in Hyperglycemia States

**DOI:** 10.3390/molecules22122249

**Published:** 2017-12-17

**Authors:** Agnieszka Szkudlarek, Danuta Pentak, Anna Ploch, Jadwiga Pożycka, Małgorzata Maciążek-Jurczyk

**Affiliations:** School of Pharmacy with the Division of Laboratory Medicine in Sosnowiec, Chair and Department of Physical Pharmacy, Jagiellońska 4, Medical University of Silesia, 41-200 Sosnowiec, Poland; dpentak@sum.edu.pl (D.P.); aploch@sum.edu.pl (A.P.); jadwiga.pozycka@o2.pl (J.P.); mmaciazek@sum.edu.pl (M.M.-J.)

**Keywords:** glycation, drug-drug-albumin binding, SFM, UV-Vis, CD and ^1^H-NMR spectroscopy

## Abstract

Serum albumin is exposed to numerous structural modifications which affect its stability and activity. Glycation is one of the processes leading to the loss of the original properties of the albumin and physiological function disorder. In terms of long lasting states of the hyperglycemia, Advanced Glycation End-products (AGEs) are formed. AGEs are responsible for cellular and tissue structure damage that cause the appearance of a number of health consequences and premature aging. The aim of the present study was to analyze the conformational changes of serum albumin by glycation—“fructation”—using multiple spectroscopic techniques, such as absorption (UV-Vis), fluorescence (SFM), circular dichroism (CD) and nuclear magnetic resonance (NMR) spectroscopy and evaluate of possible alteration of binding and competition between tolbutamide (TB, a first-generation sulfonylurea oral hypoglycemic drug) and losartan (LOS, an angiotensin II receptor (AT_1_) blocker used in hypertension (1st line with a coexisting diabetes)) in binding to non-glycated (HSA) and glycated (gHSA_FRC_) human serum albumin in high-affinity binding sites. The studies allowed us to indicate the structural alterations of human serum albumin as a result of fructose glycation. Changes in binding parameters, such as association $\left(K_{a}\right)$ or Stern-Volmer $\left(K_{SV}\right)$ constants suggest that glycation increases the affinity of TB and LOS towards albumin and affects interactions between them. The process of albumin glycation influences the pharmacokinetics of drugs, thus monitored pharmacotherapy is reasonable in the case of diabetes and hypertension polypharmacy. This information may lead to the development of more effective drug treatments based on personalized medicine for patients with diabetes. Our studies suggest the validity of monitored polypharmacy of diabetes and coexisting diseases.

## 1. Introduction

**Figure 1:**
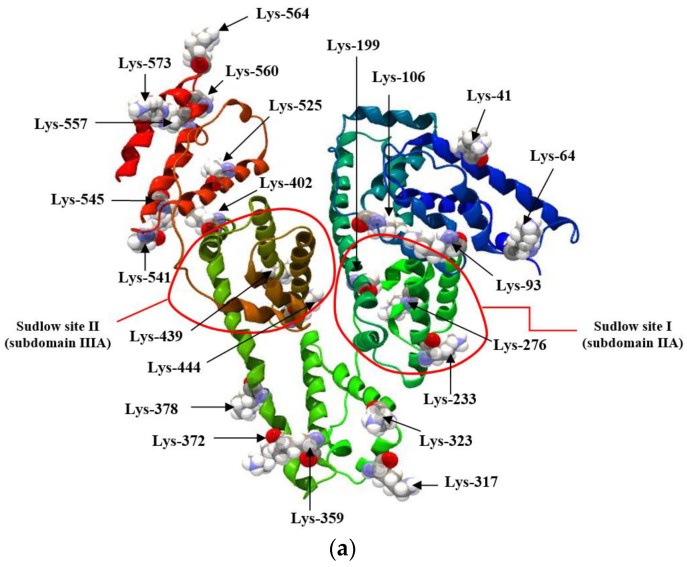

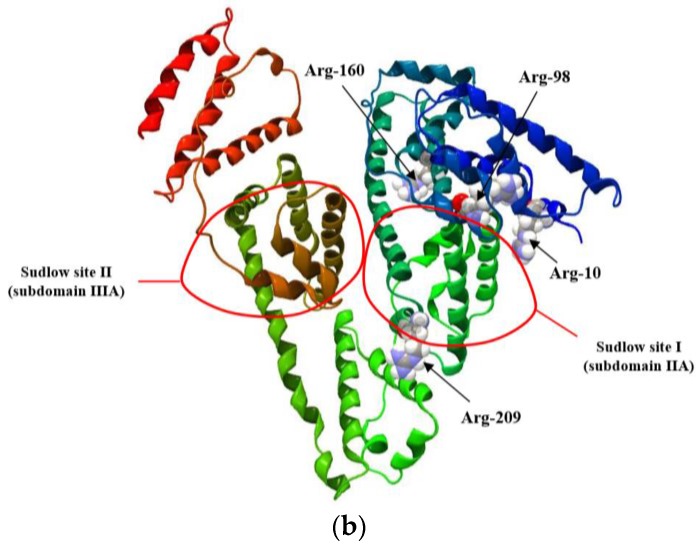
Human serum albumin drug binding sites with the location of main (**a**) lysine (Lys) and (**b**) arginine (Arg) residues involved in in vitro glycation. Molecular graphic image was produced using the CLC Drug Discovery Workbench version 1.0.2. [License: CLC-LICENSE-51JT8-DXYBY-2A3EW-ED80P-DGW80].

Human serum albumin (HSA), with a normal concentration of between 35 and 50 g/L, is the most abundant protein in plasma and has a wide variety of physiological and pharmacological functions. It is a protein reserve of the organism. HSA is responsible for maintaining normal oncotic pressure and plasma pH, transport of various endo- and exogenous ligands, as well as stabilizing the redox potential [1]. Albumin functional activity is essential for maintaining normal tissue and organ homeostasis, but its function depends on both its concentration and structure. HSA has a molecular weight of 66.7 kDa and is composed of a single polypeptide chain with 585 amino acids with one tryptophanyl residue (Trp-214), seventeen tyrosyl residues (Tyr-30, -84, -138, -140, -148, -150, -161, -263, -319, -332, -334, -341, -353, -370, -401, -411, -497), a free thiol group of a cysteinyl residue (Cys-34) and seventeen intramolecular disulfide bridges which makes it subject to a wide variety of modifications, including response to pH and other biophysical compounds [2]. HSA plays a significant role in drugs pharmacokinetics and can affect pharmacological or toxicity effect of the drug. The bound form of drug to HSA does not exhibit its pharmacodynamic effect, does not pass through biological barriers and is not subject of biotransformation, because only the free portion of the therapeutic agent shows pharmacological activity. The drug-serum albumin interactions are important component in understanding the mechanism of action and drugs distribution [3]. The capability of serum albumin to bind aromatic and heterocyclic compounds is largely dependent on the existence of two major binding regions, namely Sudlow’s site I and site II, which are located within specialized cavities in subdomains IIA and IIIA, respectively [4,5] (Figure 1). In addition, some residues, such as cysteine, lysine, serine and arginine, have found to covalently bind to many drugs [6].

It is generally believed that disorders within the spatial structure of the most important transporter proteins—the human serum albumins—induce pathological processes. The albumins with modified structures have been identified in the blood of patients with cirrhosis and chronic liver failure [7], diabetes mellitus [8,9] and also some cancers [10]. Despite the stabilization through the seventeen intra-subdomain disulfide bridges, albumin undergoes glycation that might affect the loss of physiological function. This has a crucial role in therapy planning because the nature and the strength of ligands’ interactions with their main distributor may change during the disease progression, e.g., diabetes. When protein modification induced by pathological changes occurs, an alteration of the native conformation and efficiency of these binding sites can be expected [11]. Glycation is an significant posttranslational modification of albumin that leads to formation of non-fluorescent and fluorescent Advanced Glycation End-products (AGEs), e.g., N_ε_-carboxymethyl-lysine (CML), pyrraline (Pyr), imidazolone (MG-H1, G-H1, 3-DG-H1) and pentosidine, argpyrimidine or cross-links [12,13]. Under physiological conditions most of AGEs are disintegrated and eliminated from the organism, but under pathological conditions (e.g., in hyperglycemia) glycation is more reactive. As a consequence, AGEs are accumulated and take part in the pathogenesis of many diseases related to aging and also lead to development of many diabetic complications [14]. The lysine (Lys), arginine (Arg) residues and a free thiol group of cysteinyl residue (Cys-34) located in human serum albumin are potential target for in vivo [15] and in vitro glycation, as illustrated in Figure 1.

**Figure 2:**
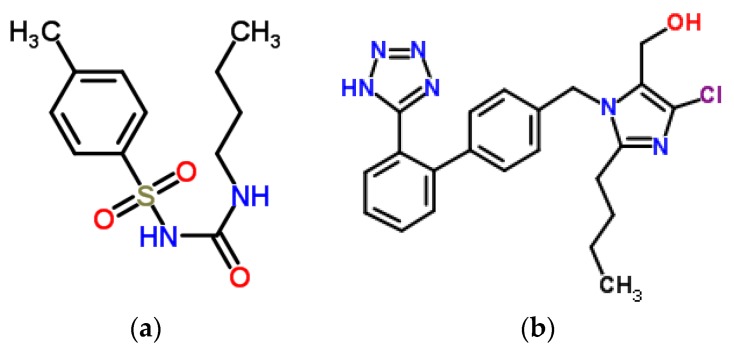
Chemical structure of (**a**) tolbutamide (TB) and (**b**) losartan (LOS).

Diabetes mellitus is a group of metabolic diseases related to increase blood sugar level. It often coexists with hypertension and arteriosclerosis which results in polypharmacy [16]. Tolbutamide (1-butyl-3-(4-methylphenylsulfonyl)urea, TB, Figure 2a) is a first-generation sulfonylurea oral hypoglycemic agent used to treat non-insulin-dependent diabetes mellitus (NIDDM). It produces hypoglycemia in adults by blocking ATP-dependent potassium (KATP) channels in pancreatic β cells resulting in insulin release. TB is metabolized by cytochrome P450 2C9 (CYP2C9) and is strongly bound to plasma protein in 91–96% [17]. Losartan ((2-butyl-4-chloro-1-{[2′-(1*H*-tetrazol-5-yl)-4-biphenylyl]methyl}-1*H*-imidazol-5-yl)methanol, LOS, Figure 2b) is an angiotensin II receptor (AT_1_) blocker used in hypertension (1st line with a coexisting diabetes) and diabetic nephropathy. LOS is a substrate of CYP2D6 and is bound with plasma protein in 98.6–98.8% [18].

Glycation may cause a number of structural changes in the spatial albumin structure which may influence the binding and cause significant drug interactions, particularly in polytherapy. The aim of the study was to investigate the influence of glycation—“fructation”—on human serum albumin structure and evaluate possible alteration of binding and competition between tolbutamide (TB) and losartan (LOS) in binding to non-glycated (HSA) and glycated (gHSA_FRC_) human serum albumin. The conformational changes of HSA cause by glycation have been analyzed by multiple spectroscopic techniques, such as fluorescence (SFM), circular dichroism (CD) and proton nuclear magnetic resonance (^1^H-NMR) spectroscopy. Binding properties of albumin due to glycation process were analyzed using absorption (UV-Vis) and fluorescence spectroscopy.

## 2. Results and Discussion

### 2.1. Effect of Glycation on Serum Albumin Tertiary Structure—Fluorescence Characteristic

**Figure 3:**
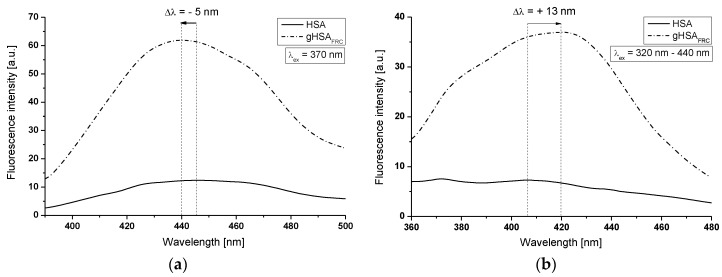
AGEs emission and synchronous fluorescence spectra of HSA and gHSA_FRC_ excited at (**a**) λ_ex_ = 370 nm and (**b**) λ_ex_ = 320–440 nm (Δλ = 40 nm), respectively; protein concentration equals to 5 × 10^−6^ mol∙L^−1^; t = 37 °C.

To prove the impact of glycation by fructose—“fructation”—on the formation of Advanced Glycation End-products (AGEs) in human serum albumin, emission and synchronous fluorescence spectra of AGEs coming from HSA and created in gHSA_FRC_ were recorded at λ_ex_ = 370 nm (Figure 3a) and at the wavelengths range λ_ex_ = 320–440 nm (Δλ = λ_em_ − λ_ex_ = 40 nm) (Figure 3b).

From the results presented in Figure 3, the higher relative fluorescence intensity obtained for gHSA_FRC_ compared with HSA indicates that the glycation products in gHSA_FRC_ are formed. After excitation at λ_ex_ = 370 nm (Figure 3a) the increase in the AGEs fluorescence intensity in gHSA_FRC_ compared to HSA was about 80% with accompanied by a blue-shift maximum fluorescence (Δλ = 5 nm). Specific emission fluorescence intensity (λ_em_ = 440 nm) observed for gHSA_FRC_ at 370 nm excitation wavelength indicates the formation of argpyrimidine (a typical fluorescent AGEs) [19]. It can be seen in Figure 3b, the marked increase in fluorescence intensity observed in glycated sample (F = 37) was about 80% compared to non-glycated HSA (F = 7.30). The main characteristic of synchronous fluorescence spectra of AGEs in gHSA_FRC_ is the red-shift maximum fluorescence (Δλ = 13 nm) from λ_em_ = 407 nm to λ_em_ = 420 nm. This miscellaneous shift of maximum fluorescence after albumin glycation (Figure 3a,b) indicates that fluorescence AGEs are chemically heterogeneous compounds. Because in the circulation, the HSA becomes glycated by reducing sugars, and the reference range of a healthy person vary between 1% and 10% [20], the blue-shift or red-shift of AGEs maximum fluorescence in gHSA_FRC_ compared with HSA makes that AGEs environment becomes more or less hydrophobic. In our previous study [21] we also observed the blue-shift of bovine serum albumin maximum fluorescence under the glycation process by fructose indicating the reduction in the polarity of AGEs environment.

**Figure 4:**
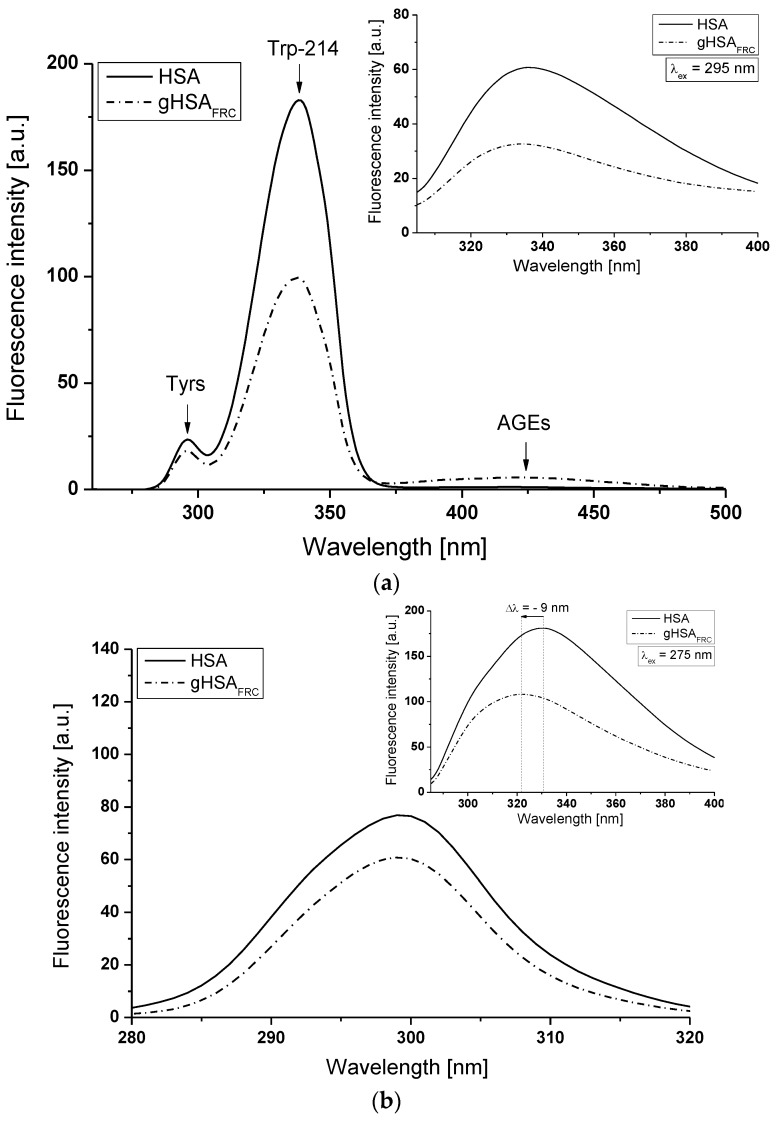
Main view: synchronous fluorescence spectra of non-glycated (HSA) and glycated (gHSA_FRC_) human serum albumin at 5 × 10^−6^ mol∙L^−1^ concentration (**a**) Δλ = 60 nm (λ_ex_ = 220–440 nm), (**b**) Δλ = 15 nm (λ_ex_ = 265–305 nm). Insert: comparison of HSA and gHSA_FRC_ emission fluorescence spectra excited at (**a**) λ_ex_ = 295 nm; (**b**) λ_ex_ = 275 nm; t = 37 °C.

Synchronous (Figure 4, main view) and emission (Figure 4, insert) fluorescence spectra of HSA and gHSA_FRC_ were used to show the conformational changes in the environment of the tryptophanyl (Trp-214) and tyrosine residues (Tyr-30, -84, -138, -140, -148, -150, -161, -263, -319, -332, -334, -341, -353, -370, -401, -411, -497) of human serum albumin influenced by glycation process. As is known, a wavelength of 275 nm excites not only Trp-214 but also tyrosine residues and it is impossible to observe separately the fluorescence of these fluorophores. Synchronous fluorescence spectroscopy allows for separation of the emission spectra originating from the Trp-214 and Tyrs (as illustrated in Figure 4a, main view), which results more specific informations about the structure of the protein. According to literature data [22,23], the synchronous fluorescence spectra were obtained considering the wavelength intervals Δλ = 60 nm and Δλ = 15 nm to evidence the Trp-214 and Tyrs, respectively (Δλ = λ_em_ − λ_ex_).

Fluorescence of HSA fluorophores is sensitive to the changes of albumin tertiary structure and environmental properties. Albumin slight structural changes near the Trp-214 and Tyrs residues affect the fluorescence intensity and position of maximum fluorescence (λ_max_) [24]. The shift of λ_max_ position corresponds to the change in polarity around the chromophore of molecule. A blue-shift of λ_max_ indicates that the amino acid residues are located in more hydrophobic environment, while a red-shift of λ_max_ implies that the Trp-214 and Tyrs residues are in a polar environment and are more exposed to the solvent [22]. Using Δλ = 60 nm (Figure 4a, main view) and Δλ = 15 nm (Figure 4b, main view), no changes in the maximum emission wavelength of HSA and gHSA_FRC_ Trp-214 and tyrosine residues were observed. It points to the stability of both bands in the synchronous spectra, irrespective of glycation process. No synchronous spectra shift caused by fructose glycation indicates no change in the polarity around Trp-214 and Tyr residues or/and a modification of the structure of human serum albumin in the environment of other residues, e.g., Cys-34. On the other hand, the main characteristic of gHSA_FRC_ emission fluorescence spectra excited at λ_ex_ = 275 nm (Figure 4b, insert) is the blue-shift maximum fluorescence (Δλ = 9 nm) from λ_em_ = 331 nm to λ_em_ = 322 nm. This phenomenon suggests that Trp-214 and Tyr residues of glycated human serum albumin are less exposed to the solvent than non-glycated macromolecule. The fluorescence intensity of both types of fluorophores in the gHSA_FRC_ spectrum is lower than in the HSA. The reduction in Trp-214 and Tyrs fluorescence intensities at λ_max_ of gHSA_FRC_ relative to HSA 45.55% (Figure 4a main view), 46.27% (Figure 4a, insert) and 20.79% (Figure 4b, main view) and 40.24% (Figure 4b, insert) have been registered. These results indicate an alteration of the albumin tertiary structure by “fructation” in the region of tryptophanyl and tyrosyl residues, which can affect the binding of drugs in subdomain IIA (Trp-214, Tyr-263), IB (Tyr-138, Tyr-140, Tyr-148, Tyr-150, Tyr-161), IIB (Tyr-319, Tyr-332, Tyr-334, Tyr-341, Tyr-353, Tyr-370) and IIIA (Tyr-401, Tyr-411, Tyr-452, Tyr-497). The loss of fluorescence intensity of Trp-214 observed for HSA glycated by glucose compared with non-glycated albumin Sakurai et al. [25] explained by energy transfer from the tryptophanyl residue to the newly chromophore formed in gHSA. Mendez et al. [26] suggested that the different fluorescence of tryptophanyl residue of non-glycated and glycated albumin can be caused due to the different hydration of the whole protein induced by glycation. Nakajou et al. [27] emphasized that an unlikely reason of the differences in fluorescence emission spectra of both albumins (non-glycated and glycated) can be directly modification of the tryptophanyl residue.

**Figure 5:**
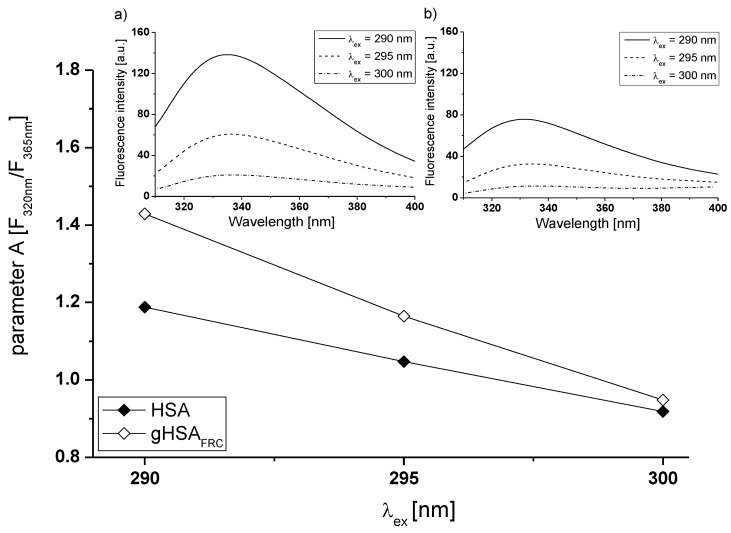
Main view: a plot of parameter $A=\frac{F_{320\;\mathrm{nm}}}{F_{365\;\mathrm{nm}}}$ vs. the excitation wavelength (λ_ex_) for HSA and gHSA_FRC_ at 5 × 10^−6^ mol∙L^−1^ concentration. Insert: emission fluorescence spectra of (**a**) HSA and (**b**) gHSA_FRC_ albumin excited at λ_ex_ = 290 nm, λ_ex_ = 295 nm and λ_ex_ = 300 nm. Error bars (error determined as a maximum deviation) are smaller than the symbols.

In order to study the structure-function relationship in proteins it is necessary to appreciate the environment and dynamics of albumin fluorophores. Red Edge Excitation Shift (REES) is an another method to directly monitor of the region surrounding the tryptophanyl residue of non-glycated and glycated human serum albumin [28,29]. In order to study REES effect, fluorescence spectra of HSA (Figure 5a, insert) and gHSA_FRC_ (Figure 5b, insert) excited at λ_ex_ = 290 nm, λ_ex_ = 295 nm and λ_ex_ = 300 nm wavelengths have been recorded. Emission fluorescence spectra of gHSA_FRC_ Trp-214 residue is different than for Trp-214 of HSA at all excitation wavelengths. A slight red-shift maximum emission of gHSA_FRC_ fluorescence (Δλ_em_ = 5 nm) relative to HSA (Δλ_em_ = 2 nm) has been obtained (Figure 5b, insert). Higher shift for glycated albumin indicates that the “fructation” of HSA decreases mobility of Trp-214 inducing changes of albumin conformation. Similarly, larger REES in case of modified-oxidized (oHSA, Δλ_em_ = 39 nm) vs. non-modified (HSA, Δλ_em_ = 4 nm) human serum albumin Maciążek-Jurczyk et al. have observed [30]. As the authors mentioned, it points to the structural modifications in the hydrophobic pocket containing the tryptophanyl residue due to the oxidation process, which contribute to stiffening of the Trp-214 environment or/and limited access to the polar solvent.

A more sensitive indicator of spectral shifts is the parameter A ($A=\frac{F_{320\;\mathrm{nm}}}{F_{365\;\mathrm{nm}}})$, which is less sensitive to experimental errors and as a consequence provides more accurate position of fluorescence spectra than in comparison with a position of the maximum fluorescence (λ_max_) [31]. In order to verify change in the fluorescence intensity of HSA and gHSA_FRC_, spectral parameter A has been calculated (Figure 5, main view). With the increase of excitation wavelength from 290 nm to 300 nm spectral parameter A decreases 1.3 times and 1.5 times for non-glycated and glycated albumin, respectively. It means that fluorescent spectra of tryptophanyl residue of HSA and gHSA_FRC_ shift towards long wavelengths (red-shift).

**Figure 6:**
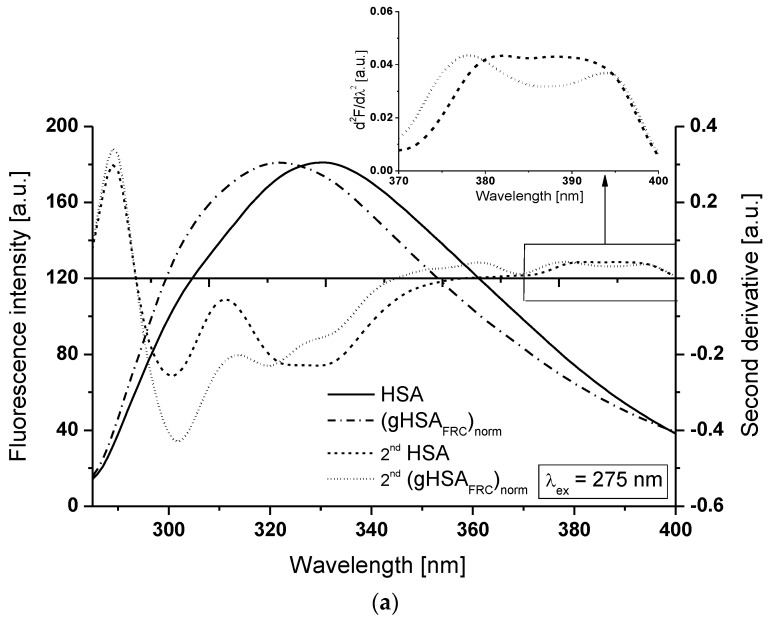

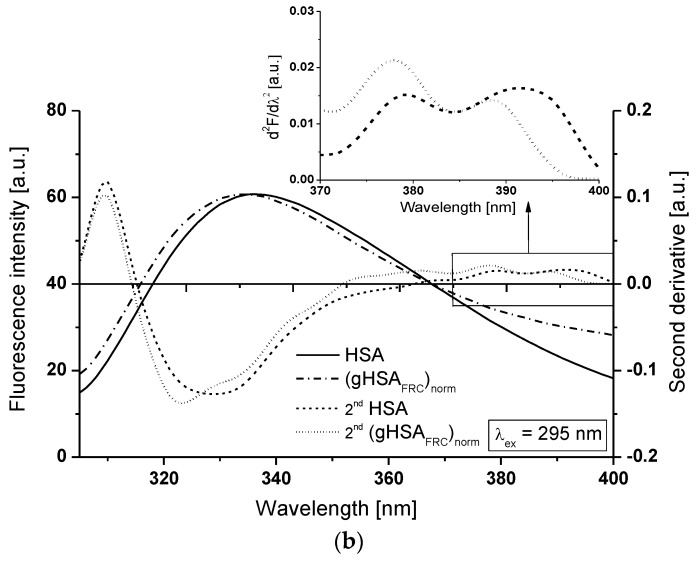
Main view: glycated albumin emission spectra normalized to non-glycated (gHSA_FRC_)_norm_ and second derivative fluorescence spectra of HSA and (gHSA_FRC_)_norm_ (2nd HSA, 2nd (gHSA_FRC_)_norm_) for (**a**) λ_ex_ = 275 nm and (**b**) λ_ex_ = 295 nm. Insert: tryptophan (Trp-214) region. The spectra were normalized to their respective maxima: λ_em_ = 331 nm (λ_ex_ = 275 nm) and λ_em_ = 336 nm (λ_ex_ = 295 nm); t = 37 °C.

In this paper, we have also used another sensitive and useful method capable of identifying subtle structural changes in the tertiary conformation of albumin caused by glycation namely second derivative of fluorescence spectra. One advantage of using this method is based on the possibility of monitoring processes in albumins, which involves relatively small changes in the environment of aromatic amino acids residues not clearly visible in classical fluorescence spectra and even in fourth derivative absorption spectroscopy [32]. Figure 6 presents the comparison of non-glycated (HSA) and glycated albumin emission spectrum normalized to non-glycated ((gHSA_FRC_)_norm_) and their second derivative fluorescence spectra (2nd HSA), (2nd (gHSA_FRC_)_norm_) for the excitation λ_ex_ = 275 nm (Figure 6a) and λ_ex_ = 295 nm (Figure 6b). The changes in the second derivative spectra in the wavelength range 370–400 nm and in the region below 320 nm point to the structure reorganization in albumin microenvironment, where tryptophan (Trp-214) and seventeen tyrosyl residues are located (in hydrophobic subdomain IIA and IB, IIB, IIA, IIIB), respectively [32,33]. It points the advantage of using the second derivative over emission fluorescence spectra, which it is more difficult to discriminate both tyrosine and tryptophan emissions [32].

At λ_ex_ = 275 nm the second derivative fluorescence spectra of non-glycated albumin (2nd HSA) exhibits three peaks maximum at wavelengths 301 nm, 324 nm and 331 nm and marked one valley at 311 nm, while the second derivative spectra of normalized glycated albumin (2nd (gHSA_FRC_)_norm_) has peaks maximum at wavelengths 302 nm and 320 nm with marked valley at 314 nm and also one shoulder from the red side of the peak at wavelength 331 nm (Figure 6a, main view). For λ_ex_ = 295 nm the second derivative fluorescence spectra of both albumins exhibit only one peak maximum at wavelengths 329 nm (for HSA) and 323 nm (for (gHSA_FRC_)_norm_) and shoulder at 333 nm (Figure 6b, main view). Hypsochromic shift of second derivative spectra of tryptophanyl (Trp-214) residue of (gHSA_FRC_)_norm_ in comparison with the second derivative fluorescence spectra of Trp-214 of HSA is observed at λ_ex_ = 275 nm (Figure 6a, insert). The second derivative spectra of Trp-214 of non-glycated albumin has only one peak maximum appear at 382 nm with a slightly marked shoulder from the red side of the peak, while the second derivative fluorescence spectra of Trp-214 of glycated albumin ((gHSA_FRC_)_norm_) exhibits two peaks maximum at 378 nm and at 394 nm. Hypsochromic shift indicates that in glycation human albumin environment around tryptophanyl residue (surrounding of subdomain IIA) becomes more hydrophobic. It confirms our previous conclusion obtained from the analysis of HSA and gHSA_FRC_ emission fluorescence spectra excited at λ_ex_ = 275 nm (Figure 4b, insert) that Trp-214 residue of glycated human serum albumin is less exposed to the solvent than tryptophanyl residue of non-glycated macromolecule. Two maxima in both second derivative fluorescence spectra of Trp-214 HSA and (gHSA_FRC_)_norm_ are observed at excitation λ_ex_ = 295 nm (Figure 6b, insert): for HSA at wavelengths 379 nm and 392 nm and for (gHSA_FRC_)_norm_ at wavelengths 378 nm and 388 nm. As illustrated in Figure 6b only second peak of Trp-214 of 2nd (gHSA_FRC_)_norm_ in comparison with Trp-214 of 2nd HSA is blue-shifted. On the contrary to the results observed for albumins excited at λ_ex_ = 275 nm (Figure 6a, insert), the more changes in the fluorescence intensities of mentioned peaks is observed for albumins excited at λ_ex_ = 295 nm (Figure 6b, insert). The use of the second derivative of the fluorescence spectra has shown that not only primarily Trp-214 located in subdomain IIA, but also Tyr residues located in subdomain IB, IIB, IIA and IIIB human serum albumin participate in the process of glycation.

In our studies we used a sensitive indicator for monitoring changes in the degree of polarity in the environment of Trp-214 and Tyr residues in both albumin (HSA and gHSA_FRC_)—empirical parameter *H* (relative peak composition) [32]. The values of parameter *H* calculated for tryptophanyl and tyrosyl residues of non-glycated (HSA) and normalized glycated (gHSA_FRC_)_norm_ albumin for λ_ex_ = 275 nm and λ_ex_ = 295 nm are collected in Table 1.

Glycation of serum albumin causes the increase in polarity around the tyrosyl (Tyr) residues (λ_ex_ = 275 nm) and the decrease in polarity around Trp-214 (λ_ex_ = 295 nm) that was shown as increase (*H*_275nm_) and decrease (*H*_295nm_) in the value of parameter *H* in (gHSA_FRC_)_norm_, respectively (Table 1). The decrease in polarity around Trp-214 in glycated human serum albumin results in blue-shift of second derivative fluorescence spectra at excitation λ_ex_ = 275 nm (Figure 6a, insert). Qualitative analysis of the second derivative spectra indicate that glycation of human serum albumin reorganizes the structure of macromolecule around Trp-214 and Tyr residues, reflecting the subtle changes in the HSA tertiary structure.

**Figure 7:**
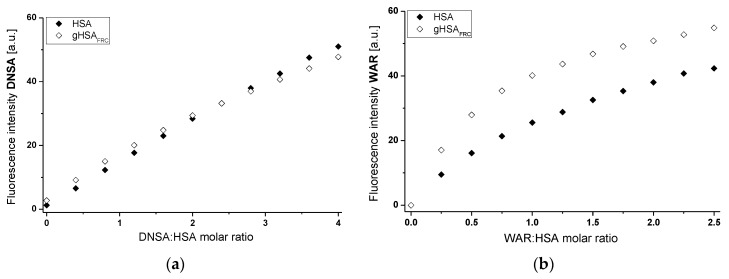

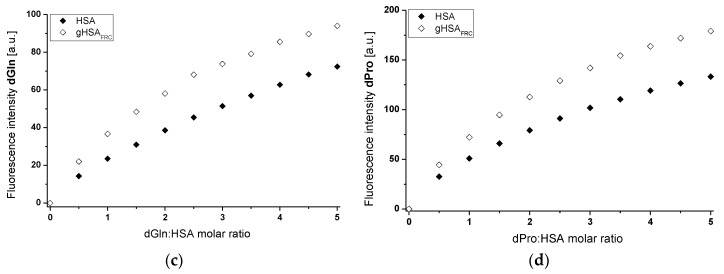
The binding capacity of fluorescent probes (**a**) DNSA, λ_ex_ = 350 nm, λ_em_ ~ 475 nm, (**b**) WAR, λ_ex_ = 330 nm, λ_em_ ~ 389 nm, (**c**) dGln, λ_ex_ = 350 nm, λ_em_ = 475 nm and (**d**) dPro, λ_ex_ = 350 nm, λ_em_ = 475 nm to non-glycated and glycated human serum albumin, protein concentration equals 5 × 10^−6^ mol∙L^−1^; t = 37 °C.

Subdomains of human serum albumin (A and B in domain I, II and III) with separate helical structures mediate albumin binding with various endogenous and exogenous ligands. There are two main binding sites for drugs in the albumin structure. According to Sudlow’s nomenclature–sites I (located in subdomain IIA) has binding affinity for heterocyclic compounds such as phentylbutazone and warfarin and site II (hydrophobic pocket in subdomain IIIA) binds to aromatic compounds such as ibuprofen [4]. Because based on the emission and synchronous fluorescence spectroscopy the changes in glycated HSA structure in the region of tryptophanyl (subdomain IIA) and tyrosyl (subdomain IB, IIB, IIA and IIIA) residues have been obtained, in order to determine the influence of glycation on hydrophobic nature of the specific binding sites, the method of fluorescent probes: 5-dimethylaminonaphthalene-1-sulfonamide (DNSA), warfarin (WAR), dansyl-l-glutamine (dGln) and *N*-dansyl-l-proline (dPro) was used. These probes do not fluoresce or exhibit weak fluorescence in the polar environment while strongly fluoresce in organic solvents (non-polar environment) or when combined with hydrophobic protein structures [34]. As seen in Figure 7 fluorescence intensity of DNSA, WAR, dGln and dPro in the complex with HSA and gHSA_FRC_ increases.

By titrating the protein solution by DNSA at increasing concentration no significant difference in the fluorescence intensity of the probe in the presence of HSA and gHSA_FRC_ has been observed (Figure 7a). In turn, by titrating the HSA and gHSA_FRC_ by warfarin solution it has been suggested that the binding of WAR with glycated HSA is stronger than with non-glycated albumin (Figure 7b). DNSA, similarly as warfarin, locates in hydrophobic regions of Sudlow’s site I in subdomain IIA [27,35]. This place consists of three subregions: Ia, Ib and Ic [36]. Registered difference in warfarin and DNSA fluorescence may result from the binding of probes in the different subregions of albumin. Based on the conducted experiment it can be concluded that glycation of HSA changes its conformation in the environment of macromolecule subdomain IIA however the magnitude of change is different for each of the Sudlow’s site I subregions. Dansyl amino acids bind to hydrophobic sites of serum albumin and dansyl-l-glutamine (dGln) was used as a marker for Sudlow’s binding site I, while *N*-dansyl-l-proline (dPro) for Sudlow’s binding site II in the HSA molecule [37]. The experiment with the dGln and dPro probes conducted a gradual increase in dGln-HSA, dGln-gHSA_FRC_ (Figure 7c) and dPro-HSA, dPro-gHSA_FRC_ (Figure 7d) fluorescence with the increase of the probe concentration. An increase in probe fluorescence, stronger for glycated than non-modified albumin, is a proof of the influence of glycation on conformation changes, both in the region of subdomain IIA, and IIIIA. Fluorescence analysis enabled the conclusion that environment of both binding site I and II is modified by fructose glycation.

### 2.2. Effect of Glycation on Human Serum Albumin Structure—Analysis of Absorption Spectra—Calculation of Free Sulfhydryl Groups Content in HSA

In order to determine a degree of glycation cysteine (Cys-34) sulfhydryl groups in non-glycated (as a control sample) and glycated human serum albumin, the Ellman’s method was used [38,39]. Cysteine sulfhydryl groups of albumin in clinical conditions are correlated with oxidative stress related chronic diseases. The number of HSA and gHSA_FRC_ free thiol group(s) was quantitatively determined with the use of 5,50-dithiobis-(2-nitrobenzoic acid) (DTNB), as a sensitive tool for determination of the oxidation of Cys residues in proteins. The thiol concentrations [SH] and the number of free sulfhydryl groups per 100 molecules of albumins—a percentage content of free sulfhydryl groups in HSA and gHSA_FRC_ [SH]% were calculated according to Equations (1) and (2), respectively. Almost four times lower thiol concentrations ($\left[{\mathrm{SH}}_{{\mathrm{gHSA}}_{\mathrm{FRC}}}\right]$ = 0.39 × 10^−6^ mol∙L^−1^, $\left[{\mathrm{SH}}_{\mathrm{HSA}}\right]$ = 1.45 × 10^−6^ mol∙L^−1^) and SH-percentage content ($\left[{\mathrm{SH}}_{{\mathrm{gHSA}}_{\mathrm{FRC}}}\right]\%$ = 7.70%, $\left[{\mathrm{SH}}_{\mathrm{HSA}}\right]\%$ = 29.10%) of glycated albumin confirm that a free thiol group of cysteine (Cys-34) located in domain I of human serum albumin are potential target for glycation.

### 2.3. Effect of Glycation on Human Serum Albumin Secondary Structure—Analysis of Circular Dichroism Spectra

**Figure 8:**
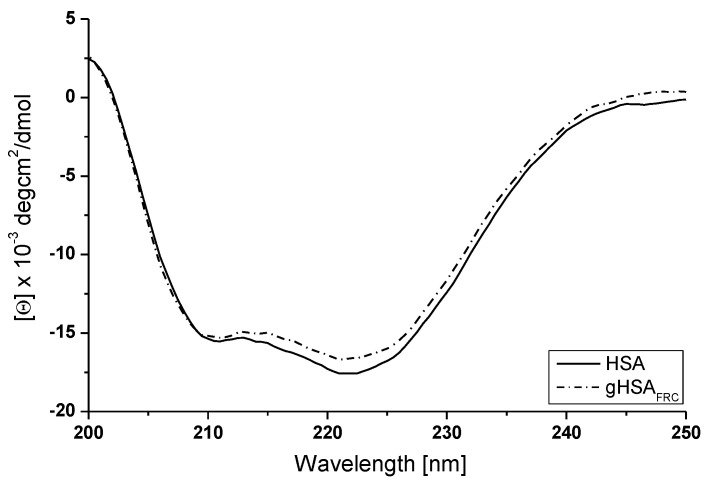
Far-UV CD spectra of non-glycated and glycated albumin (HSA, gHSA_FRC_); protein concentration equals 5 × 10^−6^ mol∙L^−1^; t = 37 °C.

The circular dichroism spectra of HSA and gHSA_FRC_ were measured in order to obtain evidence of the secondary structure changes of human serum albumin caused by glycation (Figure 8). The percentage (%) content of the secondary structure elements of non-glycated and glycated albumin are presented in Table 2.

The CD spectra of HSA and gHSA_FRC_ exhibit two negative bands in the ultraviolet region at 211 nm and 222 nm, which are characteristic of the α-helical structure of protein, because of the π-π* and n-π* transfers for the peptide bond of alpha-helix [40]. Figure 8 indicates that glycation only imperceptibly decreases both of these bands. The decrease in the band intensity of the gHSA_FRC_ at 211 and 222 nm may be indicated by the increase in the disordered structural content of albumin. Glycated albumin far-UV CD spectrum shows a decrease in the ellipticity at 222 nm in comparison to non-glycated HSA (Figure 8, Table 2). Glycation of human serum albumin causes the growth of α-helical content (about 7%) and the reduction of β-structural elements (about 4%). Similarly as in our study Trynda-Lemiesz and Wiglusz [41] reported that glycation of human serum albumin does not change considerably, about 5–6%, the overall α-helical structure of this protein.

### 2.4. Effect of Glycation on Human Serum Albumin Structure—Analysis of ^1^H-NMR Spectra

**Figure 9:**
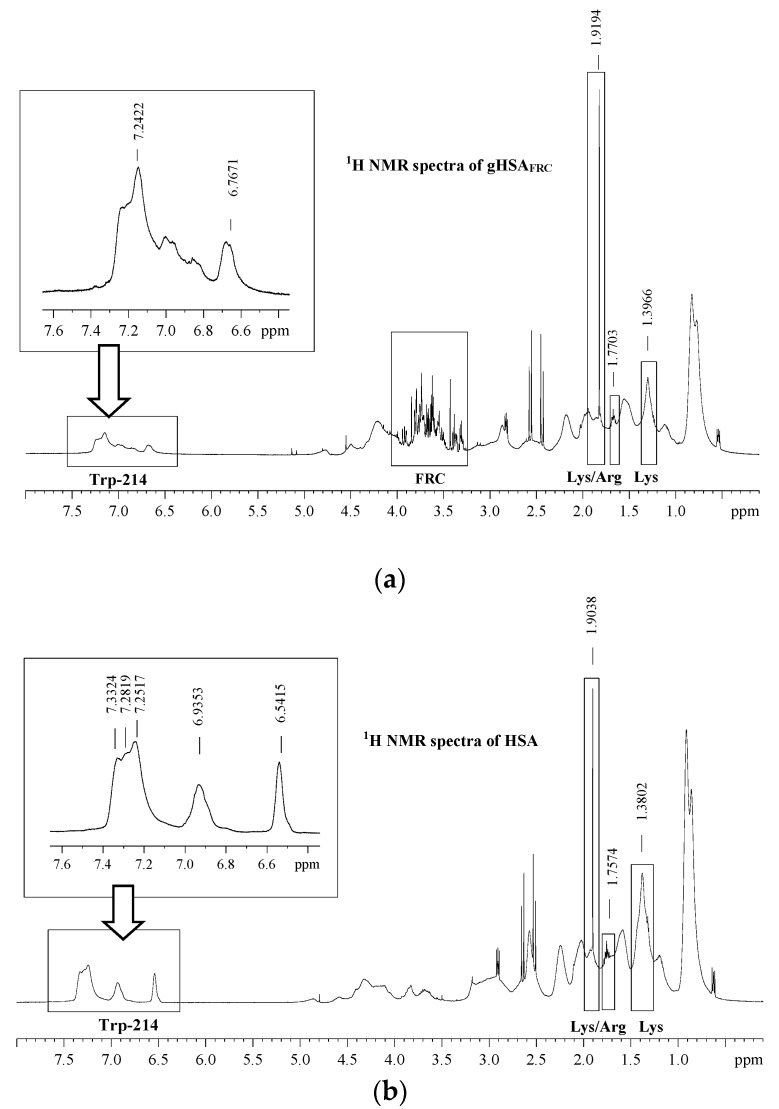
^1^H-NMR spectra of (**a**) glycated (gHSA_FRC_), (**b**) non-glycated (HSA), protein concentration equals to 1 × 10^−3^ mol∙L^−1^; t = 37 °C. ^1^H-NMR spectra of HSA come from our previous study [42].

^1^H-NMR spectra of glycated (gHSA_FRC_) (Figure 9a) and non-glycated albumin (HSA) (Figure 9b) allowed us to observe the structural changes that accompany glycations by fructose of HSA, especially in the environment of amino acid residues such as tryptophan (Trp-214), lysine (Lys) and/or arginine (Arg) residues.

On the basis of comparison of ^1^H-NMR gHSA_FRC_ and HSA spectra in aromatic and aliphatic region of albumin significant differences have been observed (Figure 9a,b). Changes in chemical shifts Δσ [ppm] of Trp-214 (Δσ = 0.0095–0.2256 ppm), Lys/Arg (Δσ = 0.0129–0.0156 ppm) and Lys (Δσ = 0.0164 ppm) proton resonances signals confirm glycation of albumin in the neighborhood of these amino acids. Changes in glycated human serum albumin structure compare to non-glycated can be explained by an upfield shift of gHSA_FRC_ resonance signals taking part in the intramolecular or intermolecular hydrogen bondings. Maciążek-Jurczyk et al. [30] emphasized that a downfield shift resonance signals of oxidized HSA (oHSA) compare to non-modified HSA points to a decrease of electron density in the surroundings of amino acid residues such as tryptophan and cysteine.

### 2.5. Fluorescence Quenching of Non-Glycated and Glycated Human Serum Albumin Induced by Tolbutamide and Losartan in the Binary and Ternary Complex

**Figure 10:**
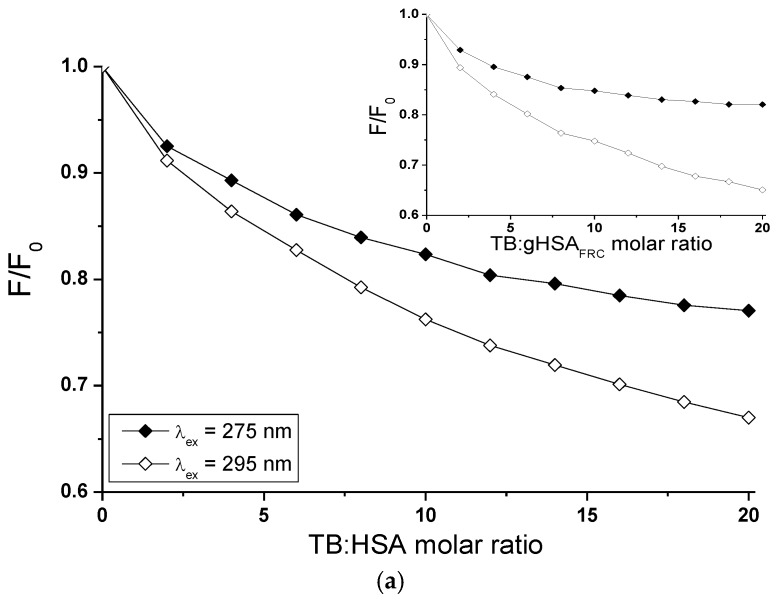

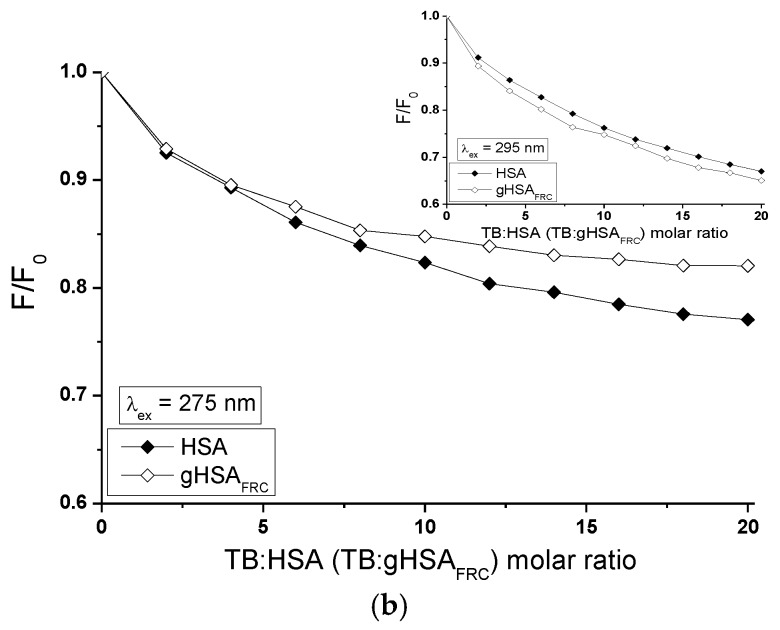
Quenching fluorescence of non-glycated (HSA) and glycated (gHSA_FRC_) human serum albumin containing 1 × 10^−5^ mol∙L^−1^–1 × 10^−4^ mol∙L^−1^ concentrations of TB. Albumin concentration: 5 × 10^−6^ mol∙L^−1^; (**a**) TB-HSA (main view, ◆) and TB-gHSA_FRC_ (insert, ◇); (**b**) λ_ex_ = 275 nm (main view, ◆) and λ_ex_ = 295 nm (insert, ◇); the error bars are smaller than the symbols.

**Figure 11:**
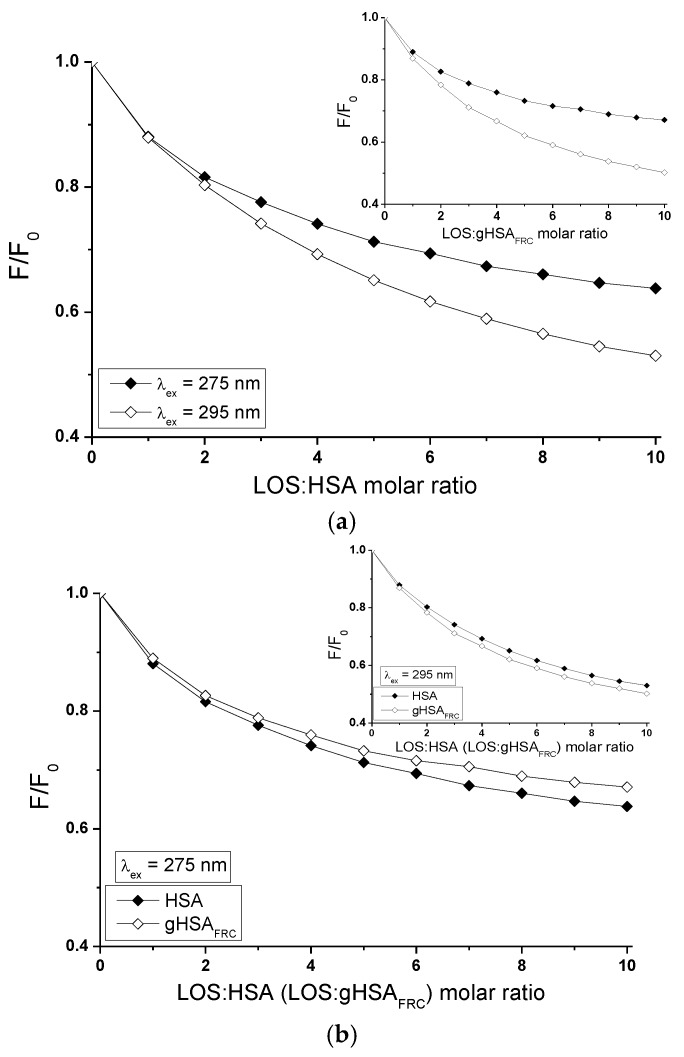
Quenching fluorescence of HSA and gHSA_FRC_ containing 5 × 10^−6^ mol∙L^−1^–5 × 10^−5^ mol∙L^−1^ concentrations of LOS. Albumin concentration: 5 × 10^−6^ mol∙L^−1^; (**a**) LOS-HSA (main view, ◆) and LOS-gHSA_FRC_ (insert, ◇); (**b**) λ_ex_ = 275 nm (main view, ◆) and λ_ex_ = 295 nm (insert, ◇); the error bars are smaller than the symbols.

Fluorescence quenching of proteins can be used to obtain detailed ligand-albumin binding informations. The fluorescence quenching of non-glycated (HSA) and glycated (gHSA_FRC_) human serum albumin excited at λ_ex_ = 275 nm and λ_ex_ = 295 nm in the binary (TB-HSA, TB-gHSA_FRC_ (Figure 10a) and LOS-HSA, LOS-gHSA_FRC_ (Figure 11a)) complexes was conducted to determine interaction of tolbutamide (TB) and losartan (LOS) in binding hydrophobic pockets with both albumins HSA and gHSA_FRC_ in the high-affinity binding sites. Glycation altered the microenvironment around tyrosyl (Tyr) and tryptophanyl (Trp-214) residues and as was described in Section 2.1 and Section 2.4, quenching curves of HSA and gHSA_FRC_ excited at λ_ex_ = 275 nm and λ_ex_ = 295 nm were compared (Figure 10b and Figure 11b). The use of the excitation wavelength of 275 nm allows to observe one tryptophanyl residue (Trp-214) and seventeen tyrosyl residues (Tyr-30, -84, -138, -140, -148, -150, -161, -263, -319, -332, -334, -341, -353, -370, -401, -411, -497) of non-glycated and glycated albumin, whereas 295 nm wavelength excites only Trp-214 of the albumins. The quenching protein fluorescence takes place when the distance between the chromophores of aromatic rings in ligand chemical structure and the fluorophores (tryptophanyl or/and tyrosyl residues) of albumin is smaller than 10 nm, typically 1–10 nm. Then, the Fluorescence Resonance Energy Transfer (FRET) donor (fluorophore)—acceptor (chromophore) is possible [43].

The quenching curves of HSA and gHSA_FRC_ in the presence of tolbutamide at increasing concentration 1 × 10^−5^ mol∙L^−1^ to 1 × 10^−4^ mol∙L^−1^ (molar ratio TB:HSA and TB:gHSA_FRC_ 2:1 to 20:1) (Figure 10a) and losartan at increasing concentration 5 × 10^−6^ mol∙L^−1^–5 × 10^−5^ mol∙L^−1^ (molar ratio LOS:HSA and LOS:gHSA_FRC_ 1:1 to 10:1) (Figure 11a) show the decrease in both non-glycated and glycated albumin fluorescence for λ_ex_ = 275 nm and λ_ex_ = 295 nm. Correction for inner filter effect has been applied (Equation (5)), so the quenching of HSA and gHSA_FRC_ fluorescence could be considered as a result of the formation of TB-HSA, TB-gHSA_FRC_ and LOS-HSA, LOS-gHSA_FRC_ complex. Tolbutamide quenches fluorescence HSA by 23% and by 33% for excitation 275 nm and 295 nm (Figure 10a, main view), respectively. Fluorescence of gHSA_FRC_ excited at λ_ex_ = 275 nm and λ_ex_ = 295 nm decreases by 18% and 35%, respectively, for the same molar ratio ligand:albumin (Figure 10a, insert). Losartan quenches fluorescence HSA by 36% and by 47%, respectively, for λ_ex_ = 275 nm and λ_ex_ = 295 nm, at molar ratio LOS:HSA 10:1 (Figure 11a, main view). Fluorescence of gHSA_FRC_ excited wavelength at 275 nm and 295 nm decreases at the same LOS:gHSA_FRC_ molar ratio by 33% and 50%, respectively (Figure 11a, insert). The quenching curves of HSA and gHSA_FRC_ excited at λ_ex_ = 275 nm and λ_ex_ = 295 nm in the presence of TB (Figure 10a) and LOS (Figure 11a) at increasing drug concentration do not overlap. This phenomenon probably means that in the interaction of tolbutamide and losartan with both serum albumins the tryptophanyl residue of subdomain IIA (Trp-214) and tyrosyl residues located in the hydrophobic subdomains i.e., IB, IIB, IIIA and IIIB take part. As seen in Figure 10a and Figure 11a, fluorescence quenching of the proteins by TB and LOS is more extended excited at 295 nm than that excited at 275 nm. The changes observed in the run of the quenching curves at λ_ex_ = 275 nm and λ_ex_ = 295 nm probably indicate significant participation of Trp-214 in the interaction between ligands (TB, LOS) and albumins (HSA, gHSA_FRC_).

The quenching curves of non-glycated and glycated albumin excited at 275 nm and 295 nm in the presence of both TB (Figure 10b) and LOS (Figure 11b) shows slight differences which originate from lower by 5% and 3% quenching of gHSA_FRC_ by TB (Figure 10b, main view) and LOS (Figure 11b, main view) and greater by 2% and 3% quenching of gHSA_FRC_ by TB (Figure 10b, insert) and LOS (Figure 11b, insert). This may be explained by structural changes around Trp-214 and Tyr microenvironment as a result of glycation.

**Figure 12:**
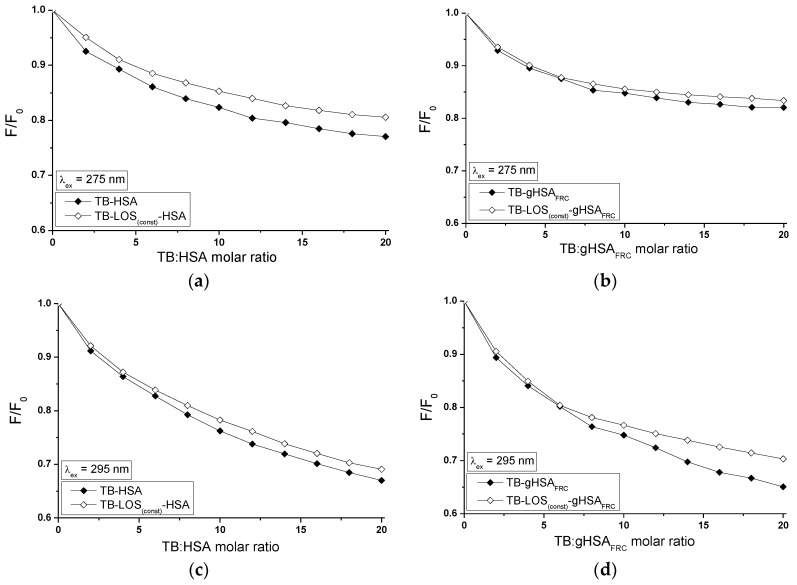
Quenching of HSA and gHSA_FRC_ fluorescence by TB and in the presence of LOS at 5 × 10^−6^ mol∙L^−1^ concentration. TB concentration varied from 1 × 10^−5^–1 × 10^−4^ mol∙L^−1^. Albumin concentration: 5 × 10^−6^ mol∙L^−1^; (**a**,**b**) λ_ex_ = 275 nm; (**c**,**d**) λ_ex_ = 295 nm; the error bars are smaller than the symbols.

**Figure 13:**
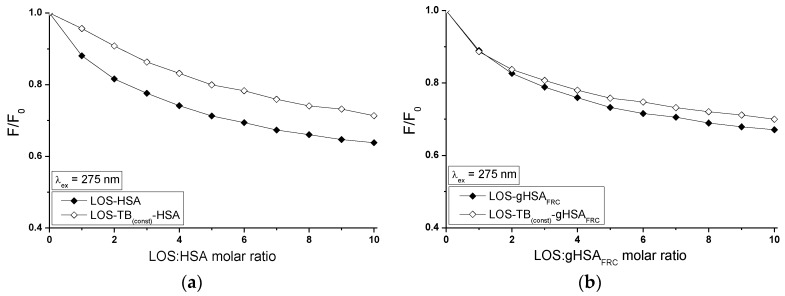

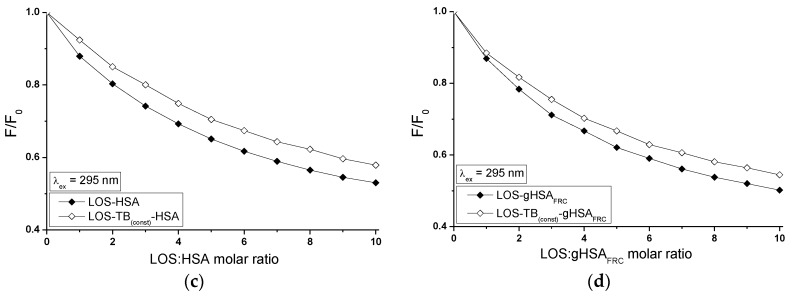
Quenching of HSA and gHSA_FRC_ fluorescence by LOS and in the presence of TB at 5 × 10^−6^ mol∙L^−1^ concentration. LOS concentration varied from 5 × 10^−6^ mol∙L^−1^–5 × 10^−5^ mol∙L^−1^. Albumin concentration: 5 × 10^−6^ mol∙L^−1^; (**a**,**b**) λ_ex_ = 275 nm; (**c**,**d**) λ_ex_ = 295 nm; the error bars are smaller than the symbols.

The influence of TB on the LOS and LOS on the TB affinity towards HSA and gHSA_FRC_ was studied by the comparison of the quenching curves of albumins (HSA, gHSA_FRC_) in the presence of TB in the binary TB-HSA, TB-gHSA_FRC_ and ternary TB-LOS_(const)_-HSA, TB-LOS_(const)_-gHSA_FRC_ (Figure 12) and in the binary LOS-HSA, LOS-gHSA_FRC_ and ternary LOS-TB_(const)_-HSA, LOS-TB_(const)_-gHSA_FRC_ complexes (Figure 13).

The quenching of fluorescence of TB-LOS_(const)_-HSA (Figure 12a,c), TB-LOS_(const)_-gHSA_FRC_ (Figure 12b,d) and LOS-TB_(const)_-HSA (Figure 13a,c), LOS-TB_(const)_-gHSA_FRC_ (Figure 13b,d) systems slightly differs from the binary TB-HSA, TB-gHSA_FRC_ and LOS-HSA, LOS-gHSA_FRC_ systems, respectively. The quenching of HSA and gHSA_FRC_ fluorescence by TB and LOS at the constant concentration is lower by 4%, 1% (for λ_ex_ = 275 nm) and 2%, 5% (for λ_ex_ = 295 nm) than in the system without additional ligand added to the binary system. The presence of LOS probably makes the interaction TB-HSA and TB-gHSA_FRC_ more difficult or hinders the formation of the TB-HSA and TB-gHSA_FRC_ complex. Losartan may probably cause displacement of TB from its complex with non-glycated and glycated serum albumin. The quenching of HSA and gHSA_FRC_ fluorescence by LOS and TB at the constant concentration is lower by 7%, 3% (λ_ex_ = 275 nm) and 5%, 4% (λ_ex_ = 295 nm) than in the binary LOS-HSA and LOS-gHSA_FRC_ systems, respectively. It shows that transfer of energy from excited fluorophores of HSA and gHSA_FRC_ to losartan is easier without tolbutamide in the system. Tolbutamide can reduce the affinity of LOS to HSA and gHSA_FRC_ because of the hydrophobic interactions stabilizing the complex TB-HSA and TB-gHSA_FRC_. It can be concluded that additional ligand changes structure of HSA and gHSA_FRC_ or/and character of binding and this may suggests that the presence of the second drug (LOS or TB) causes drug-albumin complex more stable.

**Figure 14:**
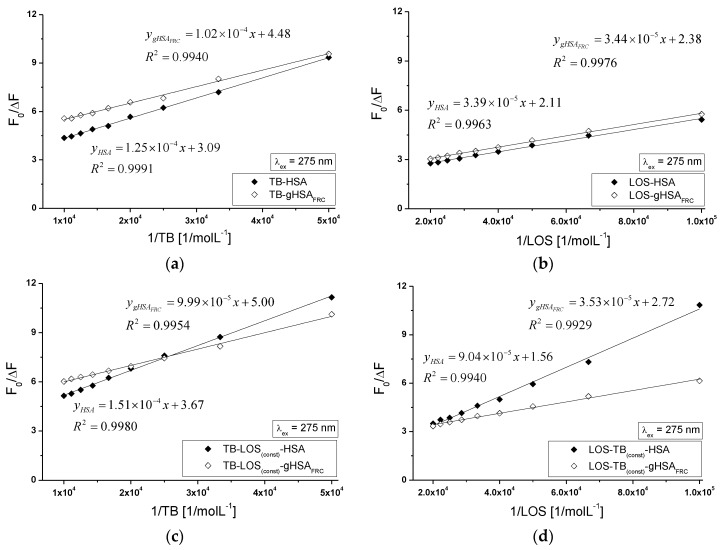
The Stern-Volmer curves modified by Lehrer for the binary (**a**) TB-HSA, TB-gHSA_FRC_; (**b**) LOS-HSA, LOS-gHSA_FRC_ and ternary systems (**c**) TB-LOS_(const)_-HSA, TB-LOS_(const)_-gHSA_FRC_, (**d**) LOS-TB_(const)_-HSA, LOS-TB_(const)_-gHSA_FRC_, λ_ex_ = 275 nm; the error bars are smaller than the symbols.

Based on the Stern-Volmer curves (Equation (6)) the mode of the interaction between tolbutamide or losartan and both serum albumin (non-glycated and glycated) in binary TB-HSA, TB-gHSA_FRC_, LOS-HSA, LOS-gHSA_FRC_ and ternary TB-LOS_(const)_-HSA, TB-LOS_(const)_-gHSA_FRC_, LOS-TB_(const)_-HSA, LOS-TB_(const)_-gHSA_FRC_ systems were analyzed (data not shown). The dependence $\frac{F_{0}}{F}$ on TB or LOS concentration when tryptophanyl (Trp-214) and 17 tyrosyl residues of the albumin have been excited displays negative deviation from the linearity. Two of the reasons is the presence of more than one fluorophore with different accessibility to the quencher (TB or LOS) and different value of the Stern-Volmer constant $K_{SV}$ (moL^−1^∙L) or the system contains a fluorophore in different environments [44,45]. The negative deviation observed in the Stern-Volmer plots is also explained in terms of intramolecular and intermolecular hydrogen bond complex formation with the fluorophore [46]. Analysis of the modified by Lehrer Stern-Volmer plots (Equation (7), Figure 14a–d) allows to determine the Stern-Volmer constant $K_{SV}$ (mol^−1^∙L) and the fractional maximum protein fluorescence accessible for the quencher $f_{a}$ for the binary (Table 3) and ternary systems (Table 4). The $K_{SV}$ is a mean value of the Stern-Volmer constants characterizing all binding sites of human serum albumin.

The Stern-Volmer constant is used to assess the availability of the quencher to the excited fluorophore. The growth of $K_{SV}$ value is associated with the increase of ligand molecule availability to the macromolecule and the formation of the complex in an excited state. The $K_{SV}$ determined on the basis of Stern-Volmer equation modified by Lehrer (Equation (7)) for binary system with non-glycated albumin (TB-HSA, LOS-HSA) is higher than that for the ternary system (TB-LOS_(const)_-HSA, LOS-TB_(const)_-HSA) while for binary system with glycated albumin (TB-gHSA_FRC_, LOS-gHSA_FRC_) $K_{SV}$ is lower than that for the ternary system (TB-LOS_(const)_-gHSA_FRC_, LOS-TB_(const)_-gHSA_FRC_) (Table 3 and Table 4). The presence of TB and LOS probably makes formation of LOS-HSA and TB-HSA complex difficult while the presence of TB and LOS in the system makes formation of LOS-gHSA_FRC_ and TB-gHSA_FRC_ complex easier, respectively. The rate of biomolecular quenching constants $k_{q}$ (10^12^) for the binary (Table 3) and ternary system (Table 4) points to the static mechanism of fluorescence quenching. By Lakowicz, the maximum value of the $k_{q}$ constant for collision fluorescence quenching in the aqueous solution equals to 2 × 10^10^ (mol^−1^∙L∙s^−1^) [47]. Static quenching leads to a decrease in the intensity of emitted fluorescence when the ligand binds to a fluorophore molecule in its basic state (non-excited) and reduces the population of fluorescents capable of excitation [48]. The Stern-Volmer values and quenching rate constants obtained for glycated albumin are higher than in comparison with $K_{SV}$ and $k_{q}$ values for non-glycated macromolecule. These results indicate that TB and LOS molecules locate closer to fluorophores of gHSA_FRC_ than HSA, both in the binary and ternary complexes. This phenomenon may probably suggests that tolbutamide (TB-HSA, TB-LOS_(const)_-HSA) and losartan (LOS-HSA, LOS-TB_(const)_-HSA) bind to non-modified albumin at such a distance that makes transfer the donor-acceptor energy difficult.

**Figure 15:**
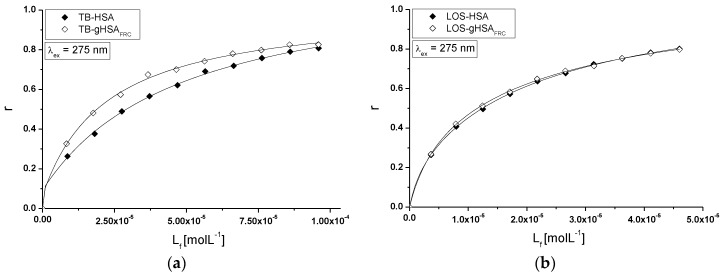

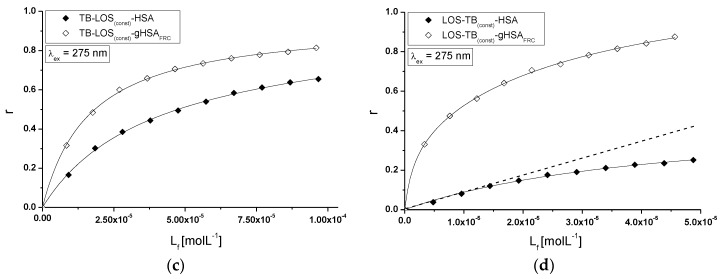
Binding isotherms of HSA and gHSA_FRC_ at 5 × 10^−6^ mol∙L^−1^ concentration with TB at 1 × 10^−5^–1 × 10^−4^ mol∙L^−1^ and LOS at 5 × 10^−6^–5 × 10^−5^ mol∙L^−1^ concentrations in the binary (**a**) TB-HSA, TB-gHSA_FRC_, (**b**) LOS-HSA, LOS-gHSA_FRC_ and ternary systems, (**c**) TB-LOS_(const)_-HSA, TB-LOS_(const)_-gHSA_FRC_ with LOS at 5 × 10^−6^ mol∙L^−1^ concentration, (**d**) LOS-TB_(const)_-HSA, LOS-TB_(const)_-gHSA_FRC_ with TB at 5 × 10^−6^ mol∙L^−1^ concentration, λ_ex_ = 275 nm; the error bars are smaller than the symbols.

A model of drug-binding to non-glycated and glycated human serum albumin in binary and ternary system has been obtained on the basis of the binding isotherms plotted based on the $r=f\left(\left[L_{f}\right]\right)$ dependence and representative data for λ_ex_ = 275 nm are presented in Figure 15. The nonlinear shape of the isotherms for TB-HSA, TB-gHSA_FRC_ (Figure 15a), LOS-HSA, LOS-gHSA_FRC_ (Figure 15b), TB-LOS_(const)_-HSA, TB-LOS_(const)_-gHSA_FRC_ (Figure 15c) and LOS-TB_(const)_-HSA, LOS-TB_(const)_-gHSA_FRC_ (Figure 15d) complexes indicates a mixed (specific and non-specific) nature of drugs interaction with both albumins in the binary and ternary system, respectively.

It means that non-specific binding sites on a HSA and gHSA_FRC_ surface, in the neighborhood of excited tyrosyl residues or/and formation of TB-HSA, TB-gHSA_FRC_, LOS-HSA, LOS-gHSA_FRC_ and TB-LOS_(const)_-HSA, TB-LOS_(const)_-gHSA_FRC_, LOS-TB_(const)_-HSA, LOS-TB_(const)_-gHSA_FRC_ complexes in hydrophobic pocket of albumin takes place.

**Figure 16:**
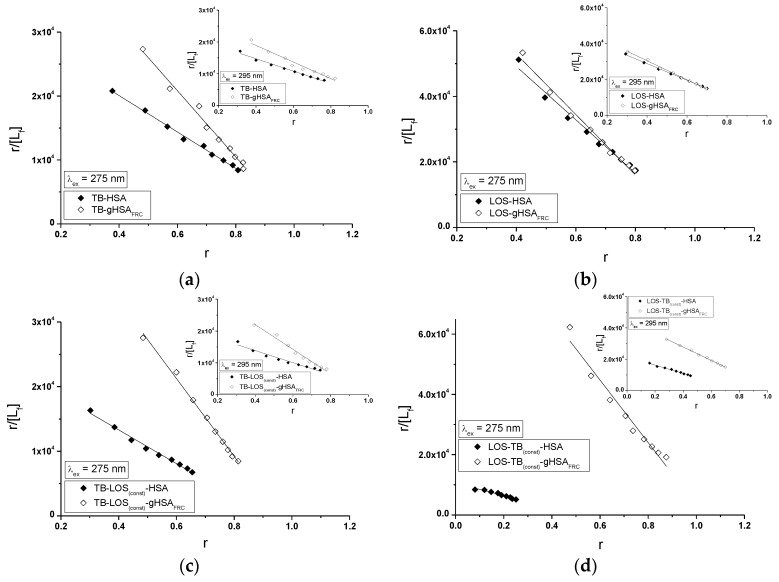
Scatchard curves of $\frac{r}{\left[L_{f}\right]}$ vs. r for the binary systems (**a**) TB-HSA, TB-gHSA_FRC_, (**b**) LOS-HSA, LOS-gHSA_FRC_ and ternary systems (**c**) TB-LOS_(const)_-HSA, TB-LOS_(const)_-gHSA_FRC_, (**d**) LOS-TB_(const)_-HSA, LOS-TB_(const)_-gHSA_FRC_; λ_ex_ = 275 nm (main view) and λ_ex_ = 295 nm (insert); the error bars are smaller than the symbols.

The Scatchard curves (the dependence of $\frac{r}{\left[L_{f}\right]}$ on r, Figure 16) and the Klotz curves (the dependence of $\frac{1}{r}$ on $\frac{1}{\left[L_{f}\right]}$, data not shown) allowed to determine association constants $K_{a}$ (mol^−1^∙L) and the number of binding sites n for the independent class of drug binding sites in albumin. The changes in high affinity binding of TB and LOS to non-glycated and glycated serum albumin in the binary and ternary systems on the basis of association constants $K_{a}$, the number of TB and LOS molecules bound with 1 mole of HSA and gHSA_FRC_ in a particular binding site n and also Hill’s coefficient $n_{H}$ cooperative obtained for the binary (TB-HSA, TB-gHSA_FRC_, LOS-HSA, LOS-gHSA_FRC_) and ternary systems (TB-LOS_(const)_-HSA, TB-LOS_(const)_-gHSA_FRC_, LOS-TB_(const)_-HSA, LOS-TB_(const)_-gHSA_FRC_) have been summarized in Table 5 and Table 6, respectively (λ_ex_ = 275 nm and λ_ex_ = 295 nm).

The Scatchard plot determined for TB-HSA, TB-gHSA_FRC_ (Figure 16a) and LOS-HSA, LOS-gHSA_FRC_ (Figure 16b) complex shows a linear dependence. This results indicates the existence of one class of specific and also non-specific binding sites on the surface of albumin for TB and LOS in HSA and gHSA_FRC_. The association constant $K_{a}$ for TB-HSA and TB-gHSA_FRC_ complex is equal to (2.84 ± 0.07) × 10^4^ mol^−1^∙L and (5.17 ± 0.23) × 10^4^ mol^−1^∙L for λ_ex_ = 275 nm and (1.94 ± 0.09) × 10^4^ mol^−1^∙L and (2.61 ± 0.19) × 10^4^ mol^−1^∙L for λ_ex_ = 295 nm, respectively (Table 5). The value of $K_{a}$ for LOS-HSA and LOS-gHSA_FRC_ complex equals to (8.13 ± 0.41) × 10^4^ mol^−1^∙L and (9.21 ± 0.40) × 10^4^ mol^−1^∙L for λ_ex_ = 275 nm and (4.62 ± 0.07) × 10^4^ mol^−1^∙L and (5.31 ± 0.11) × 10^4^ mol^−1^∙L for λ_ex_ = 295 nm, respectively (Table 6). Both TB and LOS has a high affinity towards hydrophobic subdomains IB, IIB, IIIA and IIIB of HSA due to the glycation (λ_ex_ = 275 nm) and lower, when only tryptophanyl residue was excited (λ_ex_ = 295 nm). The growth of $K_{a}$ in TB-gHSA_FRC_ and LOS-gHSA_FRC_ versus TB-HSA and LOS-HSA complexes means that in vitro glycation of albumin with lower SH-content (Section 2.2 and Section 3.3) has a higher affinity for TB and LOS compared with non-glycated albumin. In addition, losartan at 10:1 LOS:HSA (LOS:gHSA_FRC_) molar ratio has a higher affinity for HSA and gHSA_FRC_ than tolbutamide at 20:1 TB:HSA (TB:gHSA_FRC_) molar ratio. This effect shows that the transfer of energy from albumin fluorophores (Trp-214 and Tyrs) to LOS is easier than to TB. Using high-performance affinity chromatography and frontal analysis Joseph et al. studied the binding of tolbutamide to non-glycated and glycated human serum albumin at different levels of glycation [49]. The authors observed that $K_{a}$ for tolbutamide increased by 1.2- to 1.3-fold and by 1.1- to 1.4-fold in going from normal HSA to all glycated HSA at Sudlow’s site I and II, respectively. They emphasized that glycation of albumin may affect the rate of metabolism or extraction and the overall half-life of TB in the circulation. Similarly as in the previous analyzed binary systems TB-HSA, TB-gHSA_FRC_ and LOS-HSA, LOS-gHSA_FRC_, linear run of Scatchard plot for λ_ex_ = 275 nm (main view) and λ_ex_ = 295 nm (insert) indicates the existence of one class of specific and mixed, i.e., specific and non-specific binding sites for ternary system TB-LOS_(const)_-HSA, TB-LOS_(const)_-gHSA_FRC_ (Figure 16c) and LOS-TB_(const)_-HSA, LOS-TB_(const)_-gHSA_FRC_ (Figure 16d).

In order to investigate the effect of losartan and tolbutamide interactions with non-glycated and glycated albumin, the association constant $K_{a}$ for TB-HSA and TB-gHSA_FRC_ complex in the presence of LOS was carried out (Table 5). The association constants $\left(K_{a}\right)$ obtained for TB-HSA are slightly higher than that obtained for TB-LOS_(const)_-HSA ($K_{a}$ = (2.61 ± 0.10) × 10^4^ mol^−1^∙L, $K_{a}$ = (1.90 ± 0.14) × 10^4^ mol^−1^∙L), while $K_{a}$ obtained for TB-gHSA_FRC_ are slightly lower than obtained for TB-LOS_(const)_-gHSA_FRC_ ($K_{a}$= (6.03 ± 0.17) × 10^4^ mol^−1^∙L, $K_{a}$ = (3.86 ± 0.20) × 10^4^ mol^−1^∙L) complex, at both λ_ex_ = 275 nm and λ_ex_ = 295 nm. Tolbutamide has almost the same affinity to HSA than LOS_(const)_-HSA complex and to gHSA_FRC_ than LOS_(const)_-gHSA_FRC_ complex. Losartan added to the TB-HSA and TB-gHSA_FRC_ complex at 1:1 LOS:HSA and at 1:1 LOS:gHSA_FRC_ molar ratio has the abilities to form a complex with non-glycated and glycated serum albumin, where tyrosyl residues or/and tryptophanyl residue take places (in subdomain IB, IIB, IIA, IIIB or/and IIA). Because TB and LOS have albumin common binding sites, the possibility of competitive interaction between analyzed drugs should be taken into account. The number of TB molecules bound to one molecule of both HSA and gHSA_FRC_ was observed and do not change in the presence of LOS. This is similar to the results obtained for binary and ternary complexes (Table 5). Comparison of association constants calculated for binary LOS-HSA, LOS-gHSA_FRC_ and ternary complexes (LOS-TB_(const)_-HSA: $K_{a}$ = (2.10 ± 0.18) × 10^4^ mol^−1^∙L, $K_{a}$ = (2.64 ± 0.09) × 10^4^ mol^−1^∙L and LOS-TB_(const)_-gHSA_FRC_: $K_{a}$ = (10.37 ± 0.70) × 10^4^ mol^−1^∙L, $K_{a}$ = (4.26 ± 0.07) × 10^4^ mol^−1^∙L for λ_ex_ = 275 nm and λ_ex_ = 295 nm, respectively) confirms the existence of the competition between losartan and tolbutamide and the displacement of losartan from the binding site especially in non-glycated human serum albumin. It was found that the presence of TB changes the affinity of non-glycated albumin towards losartan binding site. This phenomenon is associated with the fact, that tolbutamide at 1:1 TB:HSA molar ratio interacts with tryptophanyl residue or/and tyrosyl residues located in the subdomain IB, IIB, IIA, IIIB or/and IIA, which are probably the common binding sites for both TB and LOS. Tolbutamide displaces losartan from the complex or makes the formation of LOS–HSA more difficult. The decrease in $K_{a}$ values in LOS-HSA complex due to the presence of TB means a reduction of studied system stability. The mean number of LOS molecules bound to one molecule of HSA in the given class of binding sites n is about 1 and decreased to about 0.5 for ternary LOS-TB_(const)_-HSA complex. On the other hand, glycation of albumin changes tyrosyl or/and tryptophanyl residues environment, that in the presence of TB the association constant obtained for LOS-TB_(const)_-gHSA_FRC_ is slightly higher than that obtained for LOS-gHSA_FRC_ complex at λ_ex_ = 275 nm and slightly lower at λ_ex_ = 295 nm. The mean number of LOS molecules bound to one molecule of glycated albumin in the given class of binding sites n is about 1 and does not change under the influence of the additional TB (Table 6).

The association constants $K_{a}$ (mol^−1^∙L) and the number of binding sites n (number of ligand molecules bound per protein) for the binary and ternary systems were also determined using Klotz method (the dependence of $\frac{1}{r}$ on $\frac{1}{\left[L_{f}\right]}$). The obtained results are comparable to the $K_{a}$ and n values determined using Scatchard method (Table 5 and Table 6).

In order to measure of cooperativity in a binding process, the values of the Hill coefficient $n_{H}$ has been used (Equation (5)). For TB-HSA, TB-gHSA_FRC_ and LOS-HSA, LOS-gHSA_FRC_ complex $n_{H}$ is equal to 1 or slightly more than 1. It indicates independent TB and LOS binding sites in both, non-modified and modified albumin or/and shows positive cooperativity—binding of one ligand facilitates binding of subsequent ligand at the sites on the ligand-protein complex. For ternary complexes (TB-LOS_(const)_-HSA, TB-LOS_(const)_-gHSA_FRC_, LOS-TB_(const)_-HSA, LOS-TB_(const)_-gHSA_FRC_) the Hill’s coefficients are equal to 1 and slightly less than 1 especially for LOS-TB-HSA ($n_{H}$ = 0.80 ± 0.03) and TB-LOS-gHSA_FRC_ ($n_{H}$ = 0.88 ± 0.02). This indicates negative cooperativity—binding of one ligand hinders binding of subsequent ligands at the sites on the complex.

## 3. Materials and Methods

### 3.1. Reagents

Crystallized and lyophilized human serum albumin (HSA, Lot No. 8234H) with fatty acids (fraction V) was purchased from MP Biomedicals LLC (Illkirch, France). Dansyl-l-glutamine (dGln), *N*-dansyl-l-proline (dPro), sodium azide (NaN_3_), deuterium oxide (D_2_O), tolbutamide (TB), losartan (LOS), warfarin (WAR), 5,5′-dithiobis-(2-nitrobenzoic acid) (DTNB) and 4,4-dimethyl-4-silapentane-1-sulfonic acid (DSS) were provided by Sigma-Aldrich Chemical Co. (Darmstadt, Germany). D(-)-fructose (FRC), Tris(hydroxymethyl)aminomethane pure p.a., hydrochloric acid 0.1 mol∙L^−1^ (HCl) were obtained from POCH S.A. (Gliwice, Poland). 5-Dimethylaminonaphthalene-1-sulfonamide (DNSA) was purchased from Sigma-Aldrich Chemical Co. (Buchs, Switzerland). All chemicals were of the highest analytical quality. The stock solution of dGln, dPro, TB, WAR and DNSA was prepared by dissolving appropriate amounts in methanol from Merck KGaA (Darmstadt, Germany).

### 3.2. In Vitro Modification of Human Serum Albumin

Human serum albumin (HSA) solutions at 5 × 10^−6^ mol∙L^−1^ concentration for SFM, CD, UV-Vis measurements and at 1 × 10^−3^ mol∙L^−1^ concentration for ^1^H-NMR measurements were glycated by 5 × 10^−2^ mol∙L^−1^ D(-)-fructose according to the procedure described by Szkudlarek et al. [42]. The pH 7.4 ± 0.1 of albumin solution (non-glycated (HSA) and glycated (gHSA_FRC_) was confirmed by pH meter (FEP20 Metler Toledo, Columbus, OH, USA)). The absorbance of HSA and gHSA_FRC_ at 255 nm and 280 nm ratio was less than 0.5, indicating the purity of both albumins. Modification of free thiol group in the Cys-34 residue in HSA caused by glycation was determined by the use of 5,5′-dithiobis-(2-nitrobenzoic acid) (Ellman’s reagent, DTNB). Ellman’s reagent was added to non-glycated and glycated serum albumin at 3:1 DTNB:HSA (DTNB:gHSA_FRC_) molar ratio and incubated in dark for 30 min.

### 3.3. Analysis of Absorption Spectra—Calculation of Free Sulfhydryl Groups Content in HSA

The absorption spectra of HSA, gHSA_FRC_, DTNB, DTNB-HSA and DTNB-gHSA_FRC_ complex were recorded on a model V-760 spectrophotometer (JASCO, Easton, MD, USA) equipped with a thermostat bath using 1.0 cm × 1.0 cm × 4.0 cm quartz cuvettes. The measurement range of absorption spectra of reaction mixture (DTNB-HSA, DTNB-gHSA_FRC_), protein and DTNB blank were recorded in the range 200 nm and 450 nm at 37 °C. The wavelength and photometric correction errors of the apparatus are equal to ±0.3 nm and ±0.002 Abs. at 0.5 Abs, respectively.

Sulfhydryl groups content was calculated using the molar absorption coefficient of 14,150 mol^−1^∙L∙cm^−1^ at 412 nm. The thiol molar concentrations [SH] in non-glycated and glycated albumin were determined according to Equation (1) [50]:

(1) $$\left[\mathrm{SH}\right]=\frac{Abs_{412c}-Abs_{412r}-Abs_{412p}}{\Delta \varepsilon_{412}\cdot 1cm}$$

where: $\Delta \varepsilon_{412}$ is a molar absorption coefficient at 412 nm; $Abs_{412c}$ is an absorbance of DTNB-HSA (DTNB-gHSA_FRC_) complex; $Abs_{412r}$ is an absorbance of DTNB blank; $Abs_{412p}$ is an absorbance of HSA and gHSA_FRC_ blank.

Percentage content of free sulfhydryl groups [SH]% (number of free sulfhydryl groups per 100 molecules of HSA and gHSA_FRC_, respectively) were calculated by Equation (2):

(2) $$\left[\mathrm{SH}\right]\%=\frac{\left[\mathrm{SH}\right]}{\left[\mathrm{HSA}\right]\;\mathrm{or}\;[{\mathrm{gHSA}}_{\mathrm{FRC}}]}\cdot 100$$

where: [SH] is free sulfhydryl groups molar concentration; [HSA] and $\left[{\mathrm{gHSA}}_{\mathrm{FRC}}\right]$ are the molar concentrations of non-glycated and glycated human serum albumin, respectively.

### 3.4. Preparation of Samples for Fluorescence Measurements

A quantitative analysis of dansyl-l-glutamine (dGln), *N*-dansyl-l-proline (dPro), warfarin (WAR), 5-dimethylaminonaphthalene-1-sulfonamide (DNSA) interaction with non-glycated (HSA) and glycated (gHSA_FRC_) human serum albumin, and the competition between tolbutamide (TB) and losartan (LOS) for the binding site in HSA and gHSA_FRC_ were performed by fluorimetric titration. WAR, DNSA, dGln and dPro were used as a markers for albumin Sudlow’s site I and II, respectively. To obtain dGln-HSA, dGln-gHSA_FRC_, dPro-HSA, dPro-gHSA_FRC_, DNSA-HSA, DNSA-gHSA_FRC_, WAR-HSA and WAR-gHSA_FRC_ complexes, the non-glycated and glycated albumin was titrated directly in the cuvette by the addition of increasing aliquots of dGln, dPro [2.5 × 10^−3^ mol∙L^−1^], DNSA [2 × 10^−3^ mol∙L^−1^] and WAR [1.25 × 10^−3^ mol∙L^−1^]. The HSA and gHSA_FRC_ concentration in these experiments was kept fixed at 5 × 10^−6^ mol∙L^−1^ and the molar ratio [dGln]:[HSA or gHSA_FRC_], [dPro]:[HSA or gHSA_FRC_], [DNSA]:[HSA or gHSA_FRC_] and [WAR]:[HSA or gHSA_FRC_] were varied from 0.5:1, 0.4:1 and 0.25:1 to 5:1, 4:1 and 2.5:1, respectively. To study the binding of ligand to HSA and gHSA_FRC_, tolbutamide or losartan in the absence (binary complex: TB-HSA, TB-gHSA_FRC_, LOS-HSA, LOS-gHSA_FRC_) and in the presence of LOS and TB at constant concentrations (ternary complex: TB-LOS(const)-HSA, TB-LOS_(const)_-gHSA_FRC_, LOS-TB_(const)_-HSA, LOS-TB_(const)_-gHSA_FRC_) were added to a solution of albumin. To obtain the binary complexes of TB with both albumin (non-glycated and glycated) and LOS-HSA and LOS-gHSA_FRC_, the albumin content [5 × 10^−6^ mol∙L^−1^] was titrated directly in the cuvette by the addition of increasing aliquots of TB [1 × 10^−2^ mol∙L^−1^] and LOS [5 × 10^−3^ mol∙L^−1^] from Hamilton syringe. The concentration range for TB and LOS was from 1 × 10^−5^ mol∙L^−1^ to 1 × 10^−4^ mol∙L^−1^ and from 5 × 10^−6^ mol∙L^−1^ to 5 × 10^−5^ mol∙L^−1^, respectively. To obtain the ternary complexes TB-LOS_(const)_-HSA, TB-LOS_(const)_-gHSA_FRC_ and LOS-TB_(const)_-HSA, LOS-TB_(const)_-gHSA_FRC_, the albumin content [5 × 10^−6^ mol∙L^−1^] in the presence of TB [5 × 10^−6^ mol·L^−1^] or LOS [5 × 10^−6^ mol·L^−1^] (molar ratio drug-albumin 1:1) was titrated in the cuvette by the addition of ten aliquots of TB [1 × 10^−2^ mol·L^−1^] or LOS [5 × 10^−3^ mol·L^−1^], respectively. The procedure was done directly before fluorescence measurements. The stock solutions of TB and LOS were prepared by dissolving appropriate amounts in methanol (not exceeding 1% *v/v* in the final concentration) and distilled water, respectively.

### 3.5. Fluorescence, UV-Vis Spectra and Fluorescence Second Derivative Spectra

The fluorescence measurements of the samples were recorded at 37 °C using a JASCO FP-6500 fluorescence spectrophotometer equipped with Peltier thermostat (JASCO, Easton, MD, USA) and 1.0 cm quartz cells. Excitation at λ_ex_ = 370 nm (λ_em_ = 390–500 nm) was applied for measurement of the fluorescent Advanced Glycation End-products (AGEs) in non-glycated and glycated human serum albumin. Excitation and emission slit widths were 5.0 nm. The emission fluorescence spectra of tryptophanyl (Trp-214) and thyrosyl (Tyr) residues of HSA and gHSA_FRC_ were recorded at the excitation wavelength λ_ex_ = 275 nm (λ_em_ = 285–400 nm) and the fluorescence spectra of the Trp-214 were measured at λ_ex_ = 295 nm (λ_em_ = 305–400 nm). The synchronous fluorescence spectra were obtained considering the wavelength intervals ∆λ = 15 nm, ∆λ = 60 nm and ∆λ = 40 nm to evidence the protein fluorophores (Tyr residues, Trp-214 residue) and AGEs fluorophores, respectively (∆λ—difference between emission (λ_em_) and excitation (λ_ex_) wavelength).

The loss (Equation (3)) or increase (Equation (4)) in the fluorescence intensity (F) was calculated from the following equations:

(3) $$\%\;loss\;of\;F=\left(\frac{F_{\mathrm{HSA}}-F_{{\mathrm{gHSA}}_{\mathrm{FRC}}}}{F_{\mathrm{HSA}}}\right)\cdot 100\%$$

(4) $$\%\;increase\;of\;F=\left(\frac{F_{{\mathrm{gHSA}}_{\mathrm{FRC}}}-F_{\mathrm{HSA}}}{F_{{\mathrm{gHSA}}_{\mathrm{FRC}}}}\right)\cdot 100\%$$

Red Edge Excitation Shift (REES) of HSA was compared to gHSA_FRC_ one with the use of λ_ex_ = 290 nm, λ_ex_ = 295 nm and λ_ex_ = 300 nm. The measurement range of all emission spectra were recorded from 310 nm to 400 nm and slit widths were 3.0 nm/3.0 nm. The excitation wavelength of λ_ex_ = 350 nm (λ_em_ = 400–600 nm) and λ_ex_ = 330 nm (λ_em_ = 340–500 nm) was used for measurement of dansylo-l-glutamine (dGln), *N*-dansylo-l-proline (dPro), 5-dimethylaminonaphthalene-1-sulfonamide (DNSA) and warfarin (WAR) in the complex with HSA and gHSA_FRC_, respectively.

To excite the albumin fluorophores in the binary (TB-HSA, TB-gHSA_FRC_, LOS-HSA, LOS-gHSA_FRC_) and ternary systems (TB-LOS_(const)_-HSA, TB-LOS_(const)_-gHSA_FRC_, LOS-TB_(const)_-HSA, LOS-TB_(const)_-gHSA_FRC_), λ_ex_ = 295 nm (λ_em_ = 305–400 nm) and λ_ex_ = 275 nm wavelengths (λ_em_ = 285–400 nm) were employed. Finally, light scattering caused by buffer was subtracted from fluorescence of samples in each spectrum using software supplied by JASCO (Spectra Manager).

The intensity of observed albumin fluorescence ($F_{obs}$) in the presence of losartan was corrected for the inner filter effect $\left(F_{cor}\right)$ using Equation (5) [51]. Absorbance of tolbutamide at the concentration used was below 0.05, therefore the fluorescence spectra have not been corrected.

(5) $$F_{cor}=F_{obs}\cdot e^{\left(\frac{Abs_{ex}+A_{bsem}}{2}\right)}$$

where: $F_{cor}$ and $F_{obs}$ are the fluorescence intensity corrected and observed, respectively; $Abs_{ex}$ and $Abs_{em}$ are the absorbance at excitation (λ_ex_ = 275 nm or λ_ex_ = 295 nm) and emission wavelength for HSA (λ_ex_ = 275 nm: λ_em_ = 324 nm or λ_ex_ = 295 nm: λ_em_ = 333 nm) and gHSA_FRC_ (λ_ex_ = 275 nm: λ_em_ = 321 ± 2 nm or λ_ex_ = 295 nm: λ_em_ = 333 nm), respectively. Detailed procedure: Firstly, scan the wavelength from 250 nm to 350 nm to measure the absorption of HSA, gHSA_FRC_, TB_(const)_-HSA (TB:HSA 1:1 molar ratio) and TB_(const)_-gHSA_FRC_ (TB:gHSA_FRC_ 1:1 molar ratio). Secondly, successively add losartan (5 × 10^−3^ mol∙L^−1^) to the quartz cell, once 3 μL, until the absorbance does not exceed 0.3 near excitation and emission wavelength, and record the LOS-HSA, LOS-gHSA_FRC_, LOS-TB_(const)_-HSA and LOS-TB_(const)_-gHSA_FRC_ UV-Vis absorption.

Fluorescence second derivative spectra of HSA and gHSA_FRC_ were obtained in Spectra Analysis program (version 1.53.07, JASCO, Easton, MD, USA) using Savitzky and Golay algorithm, 2nd order of polynomial and 15 data points. Then the spectra were corrected—smoothing using Savitzky and Golay method and 11 convolution width. Before the obtain of the second derivative spectra, the fluorescence spectra of gHSA_FRC_ were normalized to emission fluorescence intensities at the maximum wavelength of HSA (for λ_ex_ = 275 nm to λ_em_ = 331 nm and for λ_ex_ = 295 nm to λ_em_ = 336 nm).

### 3.6. Analysis of Fluorescence Spectra—Calculation of the Stern-Volmer and Association Constants in the Binary and Ternary Systems

Using fluorescence data, the quenching curves ($\frac{F}{F_{0}}\;$ vs. TB:HSA or TB:gHSA_FRC_ molar ratio and $\frac{F}{F_{0}}\;$ vs. LOS:HSA or LOS:gHSA_FRC_ molar ratio, *where*: F and $F_{0}$ is the fluorescence intensity at the maximum wavelength of albumin in the presence and absence of a quencher, respectively) of non-glycated and glycated human serum albumin in the presence of tolbutamide (TB) or losartan (LOS) (binary system: TB-HSA, TB-gHSA_FRC_, LOS-HSA, LOS-gHSA_FRC_) or in the presence of the second drug (ternary system: TB-LOS_(const)_-HSA, TB-LOS_(const)_-gHSA_FRC_, LOS-TB_(const)_-HSA, LOS-TB_(const)_-gHSA_FRC_) have been plotted.

The quenching effect (static and/or dynamic) of HSA, gHSA_FRC_, LOS_(const)_-HSA, LOS_(const)_-gHSA_FRC_, TB_(const)_-HSA and TB_(const)_-gHSA_FRC_ fluorescence was analyzed on the basis of the Stern-Volmer equation (Equation (6)) [52]:

(6) $$\frac{F_{0}}{F}=1+k_{q}\tau_{0}\cdot \left[L\right]=1+K_{SV}\cdot \left[L\right]$$

where: $k_{q}$ is the bimolecular quenching rate constant [mol^−1^∙L∙s^−1^]; $\tau_{0}$ is the average fluorescence lifetime of albumin without of quencher $\tau_{0}$ = 6.2 × 10^−9^ s [53]; $K_{SV}$ is the Stern-Volmer constant [mol^−1^∙L]; [L] is the ligand concentration [mol∙L^−1^]; $\left[L\right]=\left[L_{b}\right]+[L_{f}]$, $\left[L_{b}\right]$ and $\left[L_{f}\right]$ are the bound and free (unbound) drug concentrations [mol∙L^−1^].

Isotherms of drug binding to non-glycated and glycated human serum albumin in binary and ternary system have been obtained based on the graph of the function $r=f\left(\left[L_{f}\right]\right)$, where: $r=\frac{\left[L_{b}\right]}{\left[\mathrm{HSA}\right]}$ is the number of ligands moles bound per mole of protein molecule; $\left[L_{b}\right]=\frac{\Delta F}{\Delta F_{max}}\times {\mathrm{HSA}}_{total}$, ΔF is the difference between $F_{0}$ and F, $\Delta F_{max}\;$ (maximal fluorescence change with complete saturation) is evaluated from the linear part of the $\frac{1}{\Delta F}$ vs. $\frac{1}{\left[L\right]}$; [HSA] is serum albumin concentration [mol∙L^−1^] [54].

To analyze the interactions between tolbutamide and losartan and human serum albumin (HSA, gHSA_FRC_) in binary and ternary systems, the Stern-Volmer $\left(K_{SV}\right)$ and association $\left(K_{a}\right)$ constants have been determined. $K_{SV}$ constant of protein fluorescence was calculated using Stern-Volmer equation modified by Lehrer (Equation (7)) [55]:

(7) $$\frac{F_{0}}{\Delta F}=\frac{1}{\left[L\right]}\cdot \frac{1}{f_{a}}\cdot \frac{1}{K_{SV}}+\frac{1}{f_{a}}$$

where: $f_{a}$ is the fractional maximum protein fluorescence accessible for the quencher.

Association constants $\left(K_{a}\right)$ were calculated by the use of Scatchard (Equation (8)) [56] and Klotz (Equation (9)) equation [57]:

(8) $$\frac{r}{\left[L_{f}\right]}=n\cdot K_{a}-K_{a}\cdot r$$

(9) $$\frac{1}{r}=\frac{1}{n}+\frac{1}{n\cdot K_{a}\cdot [L_{f}]}$$

where: n is the number of binding sites for the independent class of drug binding sites in the albumin molecule.

Hill’s coefficient was determined on the basis of the Hill method (Equation (10)) [58]:

(10) $$\log\left(\frac{r}{1-r}\right)=n_{H}\cdot log[L_{f}]+logK_{a}$$

where: $n_{H}$ is the Hill’s coefficient.

### 3.7. Circular Dichroism (CD) Spectra

Far-UV CD spectra of non-glycated and glycated human serum albumin [5 × 10^−6^ mol∙L^−1^] were recorded using a Jasco model J-1500 CD spectropolarimeter (JASCO, Easton, MD, USA). The spectra were measured in 0.5 mm path lengths of a quartz cuvette at 37 °C in a thermostated Peltier cell holder with an accuracy of ±0.05 °C. Data were scanned from 200 nm to 250 nm at wavelength intervals of 0.5 nm, the bandwidth was set at 1.00 nm, D.I.T. 4 sec. All spectra were collected continuously at a scan speed 10 nm/min and averaged over accumulation of three spectra. Prior to calculation of the final ellipticity, HSA and gHSA_FRC_ spectra were corrected by subtraction of spectra obtained for buffer (TRIS-HCl, pH 7.4 ± 0.1) measured under identical conditions. Spectra were then smoothed using Savitzky and Golay method and 11 convolution width. CD intensity is expressed as mean residue ellipticity at wavelength λ (${\left[\theta \right]}_{mrw}$) (deg·cm^2^·dmol^−1^) according to the following equation (Equation (11)) [59]:

(11) $${\left[\theta \right]}_{mrw}=\frac{MRW\cdot \theta_{\lambda }}{10\cdot d\cdot c}$$

where: MRW is the Mean Residue Weight ($MRW_{\mathrm{HSA}}=113.87$ Da); $\theta_{\lambda }$ is the observed ellipiticity at wavelength λ (deg); is the cell-pathlength (cm); c is the protein concentration (g/cm^3^).

Content of the secondary structure elements of HSA and gHSA_FRC_ was calculated in Secondary Structure Estimation program using the Reed’s Reference model.

### 3.8. Proton Nuclear Magnetic Resonance (^1^H-NMR) Spectra

Proton nuclear magnetic resonance spectra of glycated human serum albumin were recorded at 37 °C on a FT NMR spectrometer (Bruker, Karlsruhe, Germany) using a probe tuned at 600 MHz and 5 mm tubes. Presaturation method was used for water suppression. Changes of chemical shifts of gHSA_FRC_ protons Δσ [ppm] resonances were referred to 4,4-dimethyl-4-silapentane-1-sulfonic acid (DSS) as an internal reference (DSS signal is at 0.015 ppm). ^1^H-NMR spectra gHSA_FRC_ analysis was performed using the Top Spin 3.1 software (Bruker, Karlsruhe, Germany).

### 3.9. Statistics

The results of the study were expressed as a mean ± relative standard deviation (RSD) from three independent experiments. Linear regression was analyzed using Origin version 8.5 software (Origin Northampton, MD, USA) by fitting experimental data to the corresponding equation.

## 4. Conclusions

Based on the presented data we have investigated the structural changes of serum albumin by fructose glycation and possible alteration of binding and competition between tolbutamide (TB) and losartan (LOS) in binding to non-glycated (HSA) and glycated (gHSA_FRC_) human serum albumin in high-affinity binding sites. Fructation alters the albumin tertiary structure in the region of tryptophanyl and tyrosyl residues, that affects the binding of drugs in subdomain IIA (Trp-214, Tyr-263), IB (Tyr-138, Tyr-140, Tyr-148, Tyr-150, Tyr-161), IIB (Tyr-319, Tyr-332, Tyr-334, Tyr-341, Tyr-353, Tyr-370) and IIIA (Tyr-401, Tyr-411, Tyr-452, Tyr-497). Moreover a free thiol group of cysteine (Cys-34) located in domain I of human serum albumin was also a potential target for glycation.

Binding of drugs to serum albumin is an essential factor responsible for both ligands concentration in blood and also their final destination. Because each conformational changes can cause macromolecule binding dysfunction we also analyzed drugs binding with serum albumin, especially under the influence of glycation conditions. Based on the fluorescence study we concluded that by the mixed (specific and non-specific) nature of drugs interaction, both tolbutamide and losartan interact with serum albumins in the tryptophanyl residue of subdomain IIA (Trp-214) and tyrosyl residues located in the hydrophobic subdomains i.e., IB, IIB, IIIA and IIIB, mainly in subdomain IIA, where Trp-214 is located. Glycation increases the affinity of TB and LOS towards albumin and affects interactions between them. The presence of TB and LOS probably makes formation of LOS-HSA and TB-HSA complex difficult while the presence of TB and LOS in the system makes formation of LOS-gHSA_FRC_ and TB-gHSA_FRC_ complex easier. Analyzing drug-albumin interaction especially in multidrug therapy we have also discovered the existence of the competition between losartan and tolbutamide in binding with HSA and the displacement of losartan from the binding site especially in non-glycated human serum albumin. We found that the presence of TB changes the affinity of non-glycated albumin towards losartan binding site and tolbutamide displaces losartan from the complex or makes the formation of LOS–HSA more difficult.

In summary, simultaneous use of various techniques allowed us to obtain comprehensive information about the glycation, the nature of the interaction between drugs and albumin and the possible competition between them. It can be stated with confidence that these observation may lead to the development of more effective drug treatments based on personalized medicine for patients, especially those with diabetes.

## Figures and Tables

**Table 1:** Parameter *H* calculated for Trp-214 and Tyr residues of human serum albumin, at λ_ex_ = 275 nm and λ_ex_ = 295 nm.

	*H*_275nm_	λ_min_ (nm)	λ_max_ (nm)	*H*_295nm_	λ_min_ (nm)	λ_max_ (nm)
**HSA**	0.201	301	311	0.004	385	392
**(gHSA_FRC_)_norm_**	0.228	302	314	0.002	385	389

**Table 2:** The percentage (%) content of the secondary structure elements of HSA and gHSA_FRC_ was calculated in Secondary Structure Estimation program using the Reed’s Reference model.

	${\left[\theta \right]}_{mrw}$ at 222 nm (deg·cm^2^·dmol^−1^)	% α-Helix	% β-Sheet	% Other
**HSA**	−11,770.59	36.10	47.60	16.30
**gHSA_FRC_**	−11,108.69	43.30	43.80	12.90

**Table 3:** Stern-Volmer constants $K_{SV}$ (mol^−1^∙L), fractional accessible protein fluorescence $f_{a}$ and biomolecular quenching rate constant $k_{q}$ (mol^−1^∙L∙s^−1^) calculated for the binary system: TB-HSA, TB-gHSA_FRC_, LOS-HSA, LOS-gHSA_FRC_; λ_ex_ = 275 nm and λ_ex_ = 295 nm.

**λ_ex_ = 275 nm**	$K_{SV}$ ± RSD *^)^ × 10^4^ (mol^−1^∙L)	$f_{a}$ ± RSD *^)^	**^a^** $k_{q}$ ± RSD *^)^ × 10^12^ (mol^−1^∙L∙s^−1^)
TB-HSA	2.47 ± 0.01	0.32 ± 0.01	3.98 ± 0.02
TB-gHSA_FRC_	4.39 ± 0.05	0.22 ± 0.01	7.08 ± 0.08
LOS-HSA	6.24 ± 0.02	0.47 ± 0.01	10.06 ± 0.03
LOS-gHSA_FRC_	6.92 ± 0.03	0.42 ± 0.01	11.16 ± 0.05
**λ_ex_ = 295 nm**	$K_{SV}$ ± RSD *^)^ × 10^4^ (mol^−1^∙L)	$f_{a}$ ± RSD *^)^	**^a^** $k_{q}$ ± RSD *^)^ × 10^12^ (mol^−1^∙L∙s^−1^)
TB-HSA	1.83 ± 0.02	0.50 ± 0.02	2.95 ± 0.03
TB-gHSA_FRC_	2.45 ± 0.01	0.48 ± 0.02	3.95 ± 0.02
LOS-HSA	3.71 ± 0.01	0.72 ± 0.01	5.98 ± 0.02
LOS-gHSA_FRC_	4.15 ± 0.02	0.74 ± 0.01	6.69 ± 0.03

*^)^ relative standard deviation; ^a^ calculated using: $k_{q}=\frac{K_{SV}}{\tau_{0}}$, where: $\tau_{0}$ = 6.2 × 10^−9^ s [19]—the average fluorescence lifetime of albumin without of quencher.

**Table 4:** Stern-Volmer constants $K_{SV}$ (mol^−1^∙L), fractional accessible protein fluoresce $f_{a}$ and biomolecular quenching rate constant $k_{q}$ (mol^−1^∙L∙s^−1^) calculated for the ternary system: TB-LOS_(const)_-HSA, TB-LOS_(const)_-gHSA_FRC_, LOS-TB_(const)_-HSA, LOS-TB_(const)_-gHSA_FRC_; λ_ex_ = 275 nm and λ_ex_ = 295 nm.

**λ_ex_ = 275 nm**	$K_{SV}$ ± RSD *^)^ × 10^4^ (mol^−1^∙L)	$f_{a}$ ± RSD *^)^	**^a^** $k_{q}$ ± RSD *^)^ × 10^12^ (mol^−1^∙L∙s^−1^)
TB-LOS_(const)_-HSA	2.43 ± 0.01	0.27 ± 0.01	3.92 ± 0.02
TB-LOS_(const)_-gHSA_FRC_	4.99 ± 0.06	0.20 ± 0.01	8.05 ± 0.10
LOS-TB_(const)_-HSA	1.73 ± 0.09	0.64 ± 0.05	2.79 ± 0.14
LOS-TB_(const)_-gHSA_FRC_	7.71 ± 0.09	0.37 ± 0.01	12.44 ± 0.14
**λ_ex_ = 295 nm**	$K_{SV}$ ± RSD *^)^ × 10^4^ (mol^−1^∙L)	$f_{a}$ ± RSD *^)^	**^a^** $k_{q}$ ± RSD *^)^ × 10^12^ (mol^−1^∙L∙s^−1^)
TB-LOS_(const)_-HSA	1.88 ± 0.02	0.46 ± 0.02	3.03 ± 0.03
TB-LOS_(const)_-gHSA_FRC_	3.36 ± 0.02	0.38 ± 0.01	5.42 ± 0.03
LOS-TB_(const)_-HSA	2.33 ± 0.03	0.79 ± 0.02	3.76 ± 0.05
LOS-TB_(const)_-gHSA_FRC_	3.36 ± 0.02	0.73 ± 0.01	5.42 ± 0.03

*^)^ relative standard deviation; **^a^** calculated using: $k_{q}=\frac{K_{SV}}{\tau_{0}}$, where: $\tau_{0}$ = 6.2 × 10^−9^ s [19].

**Table 5:** Association constants $K_{a}$ (mol^−1^∙L), mean number of TB molecule bound with one molecule of HSA and gHSA_FRC_ (n), the Hill’s coefficient $\left(n_{H}\right)$ in the binary (TB-HSA, TB-gHSA_FRC_) and ternary system (TB-LOS_(const)_-HSA, TB-LOS_(const)_-gHSA_FRC_); λ_ex_ = 275 nm, λ_ex_ = 295 nm.

	Scatchard Method		Klotz Method		Hill Method
**λ_ex_ = 275 nm**	$K_{a}$ ± RSD *^)^ × 10^4^ (mol^−1^∙L)	n ± RSD *^)^	$K_{a}$ ± RSD *^)^ × 10^4^ (mol^−1^∙L)	n ± RSD *^)^	$n_{H}$ ± RSD *^)^
TB-HSA	2.84 ± 0.07	1.11 ± 0.04	2.88 ± 0.01	1.10 ± 0.01	1.17 ± 0.03
TB-gHSA_FRC_	5.17 ± 0.23	1.00 ± 0.08	5.24 ± 0.10	1.00 ± 0.01	1.00 ± 0.03
TB-LOS_(const)_-HSA	2.61 ± 0.10	0.91 ± 0.05	2.73 ± 0.01	0.89 ± 0.02	0.91 ± 0.01
TB-LOS_(const)_-gHSA_FRC_	6.03 ± 0.17	0.95 ± 0.05	5.97 ± 0.06	0.95 ± 0.01	0.88 ± 0.02
**λ_ex_ = 295 nm**	$K_{a}$ ± RSD *^)^ × 10^4^ (mol^−1^∙L)	n ± RSD *^)^	$K_{a}$ ± RSD *^)^ × 10^4^ (mol^−1^∙L)	n ± RSD *^)^	$n_{H}$ ± RSD *^)^
TB-HSA	1.94 ± 0.09	1.16 ± 0.08	2.06 ± 0.02	1.12 ± 0.04	1.19 ± 0.04
TB-gHSA_FRC_	2.61 ± 0.19	1.12 ± 0.13	2.83 ± 0.01	1.09 ± 0.04	1.21 ± 0.07
TB-LOS_(const)_-HSA	1.90 ± 0.14	1.13 ± 0.12	2.13 ± 0.02	1.06 ± 0.05	1.15 ± 0.05
TB-LOS_(const)_-gHSA_FRC_	3.86 ± 0.20	0.97 ± 0.09	3.92 ± 0.04	0.96 ± 0.02	0.95 ± 0.03

*^)^ relative standard deviation.

**Table 6:** Association constants $K_{a}$ (mol^−1^∙L), mean number of LOS molecule bound with one molecule of HSA and gHSA_FRC_ (n), the Hill’s coefficient $\left(n_{H}\right)$ in the binary (LOS-HSA, LOS-gHSA_FRC_) and ternary (LOS-TB_(const)_-HSA, LOS-TB_(const)_-gHSA_FRC_) system; λ_ex_ = 275 nm, λ_ex_ = 295 nm.

	Scatchard Method		Klotz Method		Hill Method
**λ_ex_ = 275 nm**	$K_{a}$ ± RSD *^)^ × 10^4^ (mol^−1^∙L)	n ± RSD *^)^	$K_{a}$ ± RSD *^)^ × 10^4^ (mol^−1^∙L)	n ± RSD *^)^	$n_{H}$ ± RSD *^)^
LOS-HSA	8.13 ± 0.41	1.00 ± 0.08	8.64 ± 0.11	0.98 ± 0.02	1.03 ± 0.03
LOS-gHSA_FRC_	9.21 ± 0.40	0.97 ± 0.07	9.63 ± 0.08	0.96 ± 0.02	0.97 ± 0.02
LOS-TB_(const)_-HSA	2.10 ± 0.18	0.50 ± 0.06	1.85 ± 0.09	0.55 ± 0.04	0.80 ± 0.03
LOS-TB_(const)_-gHSA_FRC_	10.37 ± 0.70	1.03 ± 0.12	11.17 ± 0.27	1.01 ± 0.02	1.12 ± 0.06
**λ_ex_ = 295 nm**	$K_{a}$ ± RSD *^)^ × 10^4^ (mol^−1^∙L)	n ± RSD *^)^	$K_{a}$ ± RSD *^)^ × 10^4^ (mol^−1^∙L)	n ± RSD *^)^	$n_{H}$ ± RSD *^)^
LOS-HSA	4.62 ± 0.07	1.02 ± 0.02	4.69 ± 0.01	1.02 ± 0.01	1.03 ± 0.01
LOS-gHSA_FRC_	5.31 ± 0.11	0.97 ± 0.03	5.34 ± 0.01	0.97 ± 0.01	0.97 ± 0.01
LOS-TB_(const)_-HSA	2.64 ± 0.09	0.82 ± 0.04	2.66 ± 0.03	0.81 ± 0.02	0.90 ± 0.02
LOS-TB_(const)_-gHSA_FRC_	4.26 ± 0.07	1.05 ± 0.03	4.24 ± 0.02	1.05 ± 0.01	1.05 ± 0.01

*^)^ relative standard deviation.
